# Emergence of radial orientation selectivity: Effect of cell density changes and eccentricity in a layered network

**DOI:** 10.3389/fncom.2022.881046

**Published:** 2022-12-13

**Authors:** Catherine E. Davey, David B. Grayden, Anthony N. Burkitt

**Affiliations:** ^1^Melbourne Brain Centre Imaging Unit, University of Melbourne, Parkville, VIC, Australia; ^2^Department of Biomedical Engineering, University of Melbourne, Parkville, VIC, Australia

**Keywords:** neural network, rate-based neural plasticity, orientation selectivity, spatial opponent cells, neural learning, radial orientation

## Abstract

We establish a simple mechanism by which radially oriented simple cells can emerge in the primary visual cortex. In 1986, R. Linsker. proposed a means by which radially symmetric, spatial opponent cells can evolve, driven entirely by noise, from structure in the initial synaptic connectivity distribution. We provide an analytical derivation of Linsker's results, and further show that radial eigenfunctions can be expressed as a weighted sum of degenerate Cartesian eigenfunctions, and vice-versa. These results are extended to allow for radially dependent cell density, from which we show that, despite a circularly symmetric synaptic connectivity distribution, radially biased orientation selectivity emerges in the third layer when cell density in the first layer, or equivalently, synaptic radius, changes with eccentricity; i.e., distance to the center of the lamina. This provides a potential mechanism for the emergence of radial orientation in the primary visual cortex before eye opening and the onset of structured visual input after birth.

## 1. Introduction

Synaptic plasticity underpins our understanding of learning in neural systems as it is the mechanism that describes how synaptic weights change in response to sensory inputs. Plasticity has traditionally been modeled as rate based, in which synaptic weights change in response to short-time averaged pre- and postsynaptic neuron spiking rates. Over the past two decades, the importance of pre- and postsynaptic neuron spike timing has been recognized, particularly for contexts in which high-resolution temporal information is involved at microsecond resolution (Gerstner et al., 1996; Kempter et al., 1999a), prompting the emergence of spike-timing dependent plasticity (STDP) (Gerstner et al., 1996; Markram et al., 1997). Spike-based plasticity updates synaptic strength in response to the relative timing of pre- and postsynaptic spikes, amplifying synaptic strength if the presynaptic neuron contributes to the postsynaptic neuron's spike, and depressing a synapse if the presynaptic neuron fires after the postsynaptic neuron and thus did not contribute to its spike.

Plasticity mechanisms have played a fundamental role in explaining the emergence of simple cells in the early layers of cortical processing, such as the primary visual cortex (V1). Plasticity has successfully explained the emergence of simple cells such as orientation selective cells (Bienenstock et al., 1982; Wimbauer et al., 1998; Yamakazi, 2002), direction selective cells (Wimbauer et al., 1997a,b; Senn and Buchs, 2003), ocular dominance (Miller, 1990), and feature maps in which sensitivity to a particular feature changes as the layer is traversed (Goodhill, 2007).

Much of the research on learning in cortical networks has been empirical and computational because of the analytical complexity of learning in response to parameters that describe the number of layers, connectivity structure, and neuron type. A notable exception is the analysis of the network proposed by Linsker (1986b) in which the emergence of a spatial opponent cell in the third layer of a three-layer network of Poisson neurons with Gaussian connectivity kernels was described. Learning in this network is a linear function of correlation in presynaptic neural activity, with two learning constants that control the homeostatic equilibrium. The linearity of the learning system enables an eigenfunction analysis to be used to identify the independent contributors to a postsynaptic neuron's synaptic weight structure. Eigenvalues provide a way to distinguish the eigenfunctions that are the most significant contributors, and hence determine the receptive field of the postsynaptic neuron.

Although, Linsker (1986b) focused on empirical results, there has been significant work aimed at extending the analytical framework for the network that he proposed. MacKay and Miller (1990) proposed the first three radial eigenfunctions based on the work by Tang (1990), but without providing a derivation. The proposed eigenfunctions were for a simplified learning system in which homeostatic constants were assumed zero so that all plasticity was driven by correlation between presynaptic inputs and there was no non-competitive plasticity. They provided an empirical examination of the impact of non-zero homeostatic constants, showing that the eigenfunction of the leading eigenvalue can change in response to a change in the homeostatic equilibrium.

Miller (1990) employed Linsker (1986b) network in a model of learning in the primary visual cortex, with overlapping left and right eye inputs processed by the lateral geniculate nucleus (LGN). The network structure prompted correlation and anti-correlation in two afferents originating from either the same eye or the opposite eye, leading to the emergence of an ocular dominance feature map. Miller (1990) provided a description of an analytical derivation for the eigenfunctions of ocular dominance feature maps across the cortex.

Wimbauer et al. (1998) extended Linsker (1986b) network by incorporating lateral inhibitory connections in the third layer, showing the emergence of orientation selective cells in the third layer. They provided a derivation of Cartesian eigenfunctions for learning with homeostatic constants set to zero and empirically extended the solution to the general learning equation with non-zero homeostatic constants. They simulated the development of an orientation selective feature map distributed across the primary visual cortex using a model slightly more complex than that for which they derived the eigenfunctions.

Davey et al. (2021) relaxed Linsker (1986b) implicit assumption of homogeneous spike propagation delay between all pre- and post-synaptic connections between two layers. Distance dependent propagation delay was incorporated into all synaptic connections in the three layer network, and the consequent impact on both neural activity and synaptic plasticity analytically derived. Davey et al. (2021) established that propagation delay induces low-pass filtering by dispersing arrival times of spikes from pre-synaptic neurons, providing a natural correlation cancellation mechanism for distal connections. Cut-off frequency was found to decrease as the dendritic arbor increased in radius across the pre-synaptic layer, introducing an upper limit on temporal resolution for the network.

Analytical solutions to Linsker (1986b) learning system have played a central role in explaining the emergence of spatial opponent and orientation selective cells in the network. However, thus far, no general analytical solution has been provided, with analytical results to date being for the simplified system in which the homeostatic constants are set to zero. We provide here a solution for the eigenfunctions of Linsker (1986b) network in polar coordinates. As the system is radially symmetric, polar coordinates provide a natural coordinate system that enables an straightforward extension of polar eigenfunctions to the general learning system with non-zero homeostatic constants. One of the benefits of a full analytical solution for the network is insight into why the receptive field changes in response to changes in the homeostatic equilibrium and the framework to determine exactly when this change occurs.

Thus far, the original network proposed by Linsker (1986b) and used in the subsequent analytical analyzes of Miller (1990) and Wimbauer et al. (1998) made an assumption that cells within each layer were evenly distributed and that receptive fields of all cells in a layer were statistically identical; i.e., drawn from the same synaptic connectivity distribution. To date, there has been no exploration of the impact of relaxing this assumption. However, it is known that some biological cell layers show an uneven density of cells across the lamina and contain receptive fields with different statistical properties. For example, the retina is well known to have cell density changes as a function of eccentricity (Sjöstrand et al., 1999; Watson, 2014) and receptive field sizes of neurons in the primary visual cortex increase with stimulus eccentricity (Smith et al., 2001; Wurbs et al., 2013). Furthermore, it is well established that orientation selectivity in the primary visual cortex is biased toward radial orientation in that an orientation selective neuron in the primary visual cortex is more likely to be oriented toward the center of the retina (Rodionova et al., 2004). In this study, we explore the impact of radially dependent synaptic connection distributions on emerging receptive field properties in the third layer of Linsker (1986b) network and show how introducing radially dependent synaptic connectivity distributions in the first layer results in the emergence of radial orientation selectivity in the third layer of the network.

This paper is organized as follows. Section 2.1 introduces the network and neuron models used, based on Linsker (1986b) network. Radial eigenfunctions and eigenvalues are analytically derived for the simplified learning equation, for which the homeostatic parameters are set to zero, and then extended via perturbation analysis to the full system in Section 3. Eigenfunctions and eigenvalues are also derived in Cartesian coordinates in Section 3 and compared to the radial eigenfunctions. Finally, we show in Section 4 that the introduction of radially dependent synaptic connectivity distributions in the first layer generates radial orientation selectivity in the third layer of the network.

## 2. Methods

### 2.1. Network specification

**Figure 1:**
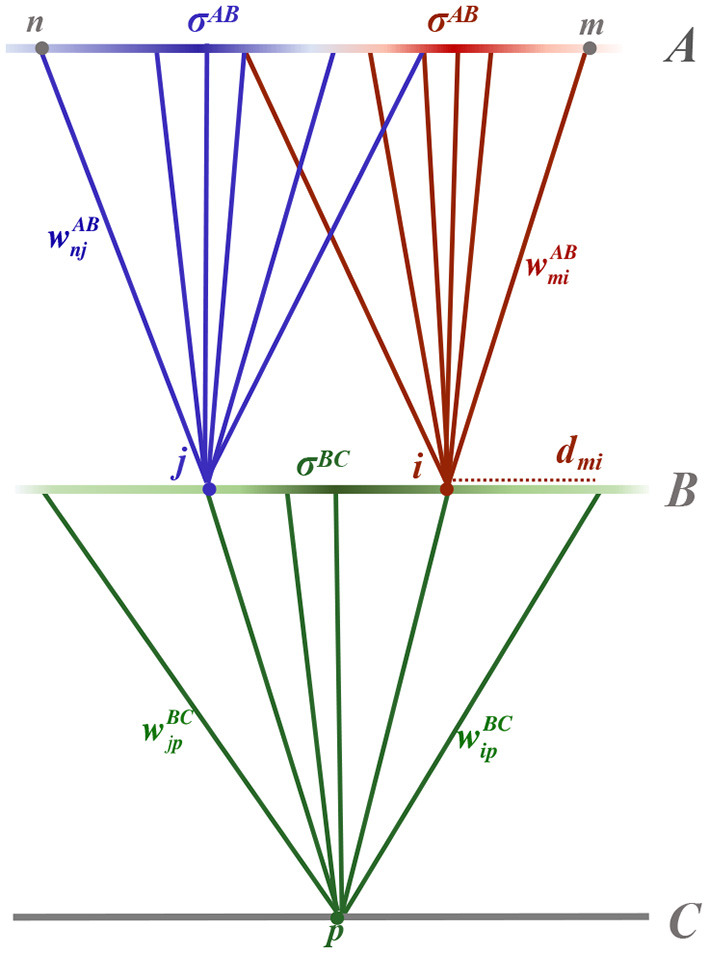
Schematic diagram of the three layered feed-forward network. Layer *A* neurons, *m* and *n*, feed into layer *B* neurons, *i* and *j*, respectively, which in turn feed to a layer *C* neuron, *p*. Synaptic connections between neurons are shown as solid, colored lines connecting from a presynaptic neuron to a postsynaptic neuron of the same color. The synaptic strength, for example between neurons *m* and *i*, is denoted $w_{mi}^{AB}$. Synaptic connection distributions are homogeneous within a layer and modeled as being Gaussian, parameterised by distance-dependent standard deviation (radius) denoted σ^*AB*^ between layers *A* and *B* and σ^*BC*^ between layers *B* and *C*. The probability of a postsynaptic neuron having a presynaptic connection from a particular position in the layer is depicted by the intensity of the color of the presynaptic layer. Not shown in the diagram is the expected number of presynaptic connections input to a neuron, denoted by *N*^*AB*^ and *N*^*BC*^ for postsynaptic neurons in layers *B* and *C*, respectively. The radial distance between two neurons within a lamina, for example between neuron *m* from layer *A* and *i* from layer *B*, is denoted $d_{mi}^{B}$.

Following Linsker (1986b), we consider a three-layer, feed-forward topographical network. The network is driven by spontaneous neural activity in the first layer, layer *A*, which inputs to layer *B*, which in turn inputs to layer *C*, as shown schematically in Figure 1. Layers comprise populations of homogeneous neurons, equispaced in a square grid across the layer. The distance between the parallel layers is assumed to dominate sufficiently such that propagation delay experienced by action potentials from the presynaptic layer can be assumed approximately equal. Neurons *m* and *n* of layer *A* have synaptic inputs to neurons *i* and *j* of layer *B*, respectively, which both input to neuron *p* of layer *C*.

Each postsynaptic neuron has a Gaussian synaptic connection distribution, centered on its two-dimensional position in the lamina, which ensures that radially proximate neurons are more likely to connect to it than a neuron more distal in the presynaptic lamina. The connectivity distributions are parameterized by a standard deviation (radius) that is homogeneous across a layer, denoted σ^*AB*^ and σ^*BC*^, for synaptic connections between layers *A* and *B* and layers *B* and *C*, respectively. Consequently, the probability of neuron *m* in layer *A* connecting to neuron *i* in layer *B* is given by

(1) $$\begin{array}{l} pN\left(\left(x_{mi},y_{mi}\right);{\left(\sigma^{AB}\right)}^{2}\right)=\frac{1}{\pi {\left(\sigma^{AB}\right)}^{2}}\exp\left(-\frac{x_{mi}^{2}+y_{mi}^{2}}{{\left(\sigma^{AB}\right)}^{2}}\right), \end{array}$$

where **x**_*mi*_ = (*x*_*mi*_, *y*_*mi*_) is the two-dimensional radial distance between *m* and *i*. Note that this definition differs from the standard definition by a factor of $\sqrt{2}$ in accordance with the definition used by Linsker (1986b), and is specifically chosen for later convenience.

For postsynaptic neurons in layer *C*, it is useful to write the connection probability in polar coordinates by assuming, without loss of generality, that the postsynaptic neuron is at position (0, 0). The probability of presynaptic neuron *j* in layer *B* connecting to postsynaptic neuron *p* in layer *C* in polar coordinates is then

(2) $$\begin{array}{l} pN\left(\left(r_{jp},\theta_{j,p}\right);{\left(\sigma^{BC}\right)}^{2}\right)=\pi {\left(\sigma^{BC}\right)}^{2}\exp\left(-\frac{r_{jp}^{2}}{{\left(\sigma^{BC}\right)}^{2}}\right), \end{array}$$

where *r*_*jp*_ is the radial distance from neuron *p*, at the center of the lamina, to neuron *j* in layer *B*, and θ_*jp*_ is the angle to *j* within the two-dimensional lamina.

Linsker (1986b) showed that the Gaussian connectivity distributions introduce spatial correlations in the inputs to layer *B* neurons despite spontaneous neural activity in layer *A* being uncorrelated. Layer *B* neurons that are spatially more proximate will have a greater number of shared connections, and therefore more correlated input, when compared to layer *B* neurons that are positioned further apart in the lamina. The expected number of shared presynaptic inputs between two postsynaptic neurons in layer *B*, denoted *N*^*BB*^, is shown to be (see Appendix A for full derivation)

(3) $$\begin{array}{l} \text{E}\left[N^{BB}\left(d_{ij}^{B}\right)\right]=\frac{{\left(N^{AB}\right)}^{2}}{2\pi {\left(\sigma^{AB}\right)}^{2}}\exp\left(-\frac{{\left(d_{ij}^{B}\right)}^{2}}{2{\left(\sigma^{AB}\right)}^{2}}\right), \end{array}$$

where *N*^*AB*^ denotes the expected number of synaptic connections from layer *A* to each neuron in layer *B*, and $d_{ij}^{B}$ represents the distance between neurons *i* and *j* such that $d_{ij}^{B}=\sqrt{x_{ij}^{2}+y_{ij}^{2}}$.

### 2.2. Neuron model

The network is driven by spontaneous Poisson activity of the layer *A* neurons. This implies that there are no spike-based temporal correlations between input and output neurons other than what is captured in the rate-based signals and that the rates change slowly when compared to the period over which they are averaged (Kempter et al., 1999b). Activity of a layer *A* neuron is modeled as $f_{m}^{A}\left(t\right)\sim Poisson(\chi^{A})$, where $f_{m}^{A}\left(t\right)$ is the spiking rate of layer *A* neuron *m* at time *t*.

As in Linsker (1986b), we use a Poisson neuron model so that the network is linear when operating within the weight bounds, discussed below. The update equations for neural activity in layers *B* and *C* are


(4a) $$\begin{array}{l} f_{i}^{B}\left(t\right)=R_{a}^{B}+\underset{m}{\sum }w_{mi}^{AB}\left(t\right)f_{m}^{A}\left(t\right), \end{array}$$

(4b) $$\begin{array}{l} f_{p}^{C}\left(t\right)=R_{a}^{C}+\underset{i}{\sum }w_{ip}^{BC}\left(t\right)f_{i}^{B}\left(t\right), \end{array}$$

where $R_{a}^{B}$, $R_{a}^{C}$ denote spontaneous firing rates, and $w_{mi}^{AB}\left(t\right)$, $w_{ip}^{BC}\left(t\right)$ depict synaptic strengths between neurons *m* and *i* in layers *A* and *B*, respectively, and neurons *i* and *p* in layers *B* and *C*, respectively. Note that an implicit assumption in this Poisson model of neural activity is that propagation delay is negligible or, equivalently, is dominated by inter-layer distances between neurons and, therefore, can be considered homogeneous across all inputs to a postsynaptic neuron.

### 2.3. Learning dynamics

The adiabatic approximation in neural learning is that incremental weight changes occur slowly with respect to neural dynamics, which occur on a millisecond timescale. Furthermore, neurons within the same population are assumed to have the same statistical properties of neural activity and synaptic connectivity. Consequently, the system is ergodic and the spike rate can be determined from the ensemble average or from a temporal mean over the timescale of learning. Under these assumptions, the learning equation can be expressed as a differential equation (Linsker, 1986b). The general learning equations for synaptic weights between neurons in layers *A* and *B* and synapses connecting layers *B* and *C* is given by Linsker (1986b)


(5a) $$\begin{array}{c} \eta {ẇ}_{mi}^{AB}=k_{1}^{AB}+\frac{1}{N^{AB}}\underset{n}{\sum }w_{ni}^{AB}\left(Q_{mn}^{A}+k_{2}^{AB}\right),\\ \;w_{\text{min}}\leq w_{mi}^{AB}\leq w_{\text{max}}, \end{array}$$

(5b) $$\begin{array}{c} \eta {ẇ}_{ip}^{BC}=k_{1}^{BC}+\frac{1}{N^{BC}}\underset{j}{\sum }w_{jp}^{BC}\left(Q_{ij}^{B}+k_{2}^{BC}\right),\\ \;w_{\text{min}}\leq w_{ip}^{BC}\leq w_{\text{max}}, \end{array}$$

where η ≪ 1 is the learning rate that ensures that learning is slow on a millisecond timescale, *w*_min_ and *w*_max_ are the lower and upper bounds on the weights, respectively, and the parameters $k_{1}^{AB}$, $k_{2}^{AB}$, $k_{1}^{BC}$, $k_{2}^{BC}$ are layer specific constants controlling homeostasis (i.e., independent of the correlation structure of the inputs). The definition for normalized covariance has the same structure for each layer; for example, the normalized covariance $Q_{mn}^{A}$ between layer *A* neurons *m* and *n* is defined by

(6) $$\begin{array}{l} Q_{mn}^{A}=\frac{\langle f_{m}^{A}-\bar{f^{A}}\rangle \langle f_{n}^{A}-\bar{f^{A}}\rangle }{f_{0}^{2}}, \end{array}$$

where 〈〉 depicts the ensemble average, $\bar{f^{A}}=\chi^{A}$, denotes the temporal average of layer *A* neural activity, and $f_{0}^{2}$ is a scaling factor to normalize the covariance matrix *Q*.

For a Gaussian synaptic density distribution, the covariance between layer *B* neurons is a function of the radial distance separating the neurons, as described in Appendix B,

(7) $$\begin{array}{l} \text{cov}\left(f_{m}^{A},f_{n}^{A}\right)=\frac{{\left(N^{AB}\bar{f^{A}}\right)}^{2}}{2\pi {\left(\sigma^{AB}\right)}^{2}}\exp\left(-\frac{{\left(d_{mn}^{A}\right)}^{2}}{2{\left(\sigma^{AB}\right)}^{2}}\right). \end{array}$$

Only radial distances are considered, so that distances between layers are assumed to have negligible impact on learning dynamics since the inter-layer transmission delay is uniform.

Normalizing this result and incorporating it into Equation (5) gives the learning equation

(8) $$\begin{array}{l} \eta {ẇ}_{mi}=k_{1}+\frac{1}{N}\underset{n}{\sum }w_{ni}\left(\exp\left(-\frac{{\left|{\text{x}}_{mi}-{\text{x}}_{ni}\right|}^{2}}{2{\left(\sigma^{AB}\right)}^{2}}\right)+k_{2}\right), \end{array}$$

where it is assumed that the covariance is normalized and we have removed the layer superscripts for readability.

It is assumed that a deeper layer is not learned until after its presynaptic layer has converged to a stable weight structure, and hence layers are learned sequentially. This accords with the approach employed by Linsker (1986b) and does not impact the final weight structure across the network. Consequently, synapses connecting layers *A* and *B* evolve to a stable structure before learning begins for synapses connecting layers *B* and *C*.

Linsker (1986b) demonstrated that individual synapses are unstable and, for excitatory synapses, all or all-but-one necessarily reach the upper bound, *w*_max_. However, under an assumption of weak covariance of the inputs (MacKay and Miller, 1990), the mean weight of synapses input to a postsynaptic neuron is not necessarily unstable but rather controlled by homeostatic mechanisms. For excitatory connections, the mean weight of a postsynaptic neuron's synapses will converge to

(9) $$\begin{array}{l} \bar{w}=-\frac{k_{1}}{k_{2}},\;\text{if}\;k_{2}<0,\text{and}\;0<\frac{k_{1}}{k_{2}}<1, \end{array}$$

where the conditions on *k*_1_ and *k*_2_ are required to ensure that the mean synaptic weight does not diverge to the bounds. For all synapses to grow until they reach the upper bound, it is required that *k*_1_ + *k*_2_ > 0. In this case, the system is unstable so that the mean synaptic weight grows until all individual synapses, or all-but-one, have reached the upper bound (Linsker, 1986b).

Linsker (1986b) selected homeostatic constants for synapses connecting layers *A* and *B* such that the mean weight was unstable and, consequently, all synapses diverged to the upper bound. For connections between layers *B* and *C*, the homeostatic constants are chosen such that the mean weight is stable, requiring some individual synapses to diverge to the lower bound and others to the upper bound.

With synaptic connections between layers *A* and *B* assumed to all reach the upper bound, the focus is on determining the learned synaptic structure for postsynaptic neurons in layer *C*. Given that the learning equation in Equation (5b) is linear within the weight bounds, the system lends itself to an eigenfunction analysis. That is, we wish to identify the independent eigenfunctions that contribute to the evolution of synaptic weights. Given that the system is driven by unstructured noise, it will self-organize such that the eigenfunction with the leading eigenvalue will ultimately dominate the synaptic weight structure.

In order to conduct an eigenfunction analysis, we approximate the discrete grid of neurons by its continuous limit. The probability of a synaptic connection existing between neuron *m* at position (*x*_*mi*_, *y*_*mi*_) in the presynaptic layer and postsynaptic neuron *i*, detailed in Equation (1), becomes a synaptic density describing the expected proportion of the total number of presynaptic inputs originating from (*x*_*mi*_, *y*_*mi*_). The synaptic strength is then considered to be the average weight of synapses at this location. In the continuous limit, the learning equation in Equation (8) becomes

(10) $$\begin{array}{l} \eta w\left(\text{x}\right)=k_{1}+\overset{\infty }{\underset{-\infty }{\int }}A\left(\exp(-\frac{{\left|\text{x}-{\text{x}}^{\prime }\right|}^{2}}{2{\left(\sigma^{AB}\right)}^{2}})+k_{2}\right)\\ \exp-(\frac{{\left|{\text{x}}^{\prime }\right|}^{2}+{\left|\text{x}\right|}^{2}}{{\left(\sigma^{BC}\right)}^{2}})w\left({\text{x}}^{\prime }\right)d^{2}{\text{x}}^{\prime }, \end{array}$$

where neuron *i* in layer *B* is denoted by its continuous position vector **x** = (*x*_*ip*_, *y*_*ip*_) and neuron *j* in layer *B* is represented by its continuous vector, ${\text{x}}^{\prime }=\left(x_{jp},y_{jp}\right)$, where vector subscripts have been omitted for readability. The Cartesian coordinates have been centered on the layer *C* neuron. Note that *A* contains coefficients to normalize covariance and connection probabilities, such that *A* = (π(σ^*BC*^)^2^)^−2^.

To characterize the system in terms of its eigenfunctions, we need to solve the eigenvalue problem for the system,

(11) $$\begin{array}{l} \lambda \eta w\left(\text{x}\right)=\overset{\infty }{\underset{-\infty }{\int }}A\left(\exp(-\frac{{\left|\text{x}-{\text{x}}^{\prime }\right|}^{2}}{2{\left(\sigma^{AB}\right)}^{2}})+k_{2}\right)\\ \;\exp-(\frac{{\left|{\text{x}}^{\prime }\right|}^{2}+{\left|\text{x}\right|}^{2}}{{\left(\sigma^{BC}\right)}^{2}})w\left({\text{x}}^{\prime }\right)d^{2}{\text{x}}^{\prime }. \end{array}$$

## 3. Radial eigenfunctions of the learning equation

Given the circular symmetry of the spatial opponent neurons that emerge from Linsker (1986b) network, we derive the radial eigenfunctions and eigenvalues of a layer *C* neuron's receptive field. By identifying the eigenfunction with the largest eigenvalue, we can analytically determine the expected receptive field of the neuron, since this eigenfunction is expected to grow most rapidly and dominate development of the receptive field.

### 3.1. Radial eigenfunctions of the simplified learning equation

To proceed we initially set *k*_2_ to zero and later consider the more general case in which *k*_2_ is non-zero. Converting to polar coordinates, such that *r* and θ give the magnitude and phase of **x**, and transforming *r* to be unit-less by scaling it by $\frac{1}{\sigma^{AB}}$, the eigenvalue problem in Equation (11) becomes

(12) $$\begin{array}{l} \lambda \eta w\left(r,\theta \right)=A{\left(\sigma^{AB}\right)}^{2}\exp-(\frac{r^{2}}{2}\left(\frac{2{\left(\sigma^{AB}\right)}^{2}+{\left(\sigma^{BC}\right)}^{2}}{{\left(\sigma^{BC}\right)}^{2}})\right)\\ \;\overset{\infty }{\underset{0}{\int }}d\tilde{r}\tilde{r}\exp-(\frac{{\tilde{r}}^{2}}{2}\left(\frac{2{\left(\sigma^{AB}\right)}^{2}+{\left(\sigma^{BC}\right)}^{2}}{{\left(\sigma^{BC}\right)}^{2}})\right)\\ \;\overset{2\pi }{\underset{0}{\int }}d\tilde{\theta }\exp\left(-\frac{-2r\tilde{r}\cos\left(\theta -\tilde{\theta }\right)}{2}\right)w\left(\tilde{r},\tilde{\theta }\right). \end{array}$$

The eigenfunctions and eigenvalues for the simplified learning equation are derived in Appendix C.1. Introducing a radial decay parameter that controls the rate of decay from the center of the receptive field,

(13) $$\begin{array}{l} C=\frac{{\left(\sigma^{BC}\right)}^{2}}{2\sigma^{AB}\sqrt{{\left(\sigma^{AB}\right)}^{2}+{\left(\sigma^{BC}\right)}^{2}}}, \end{array}$$

the eigenfunctions and associated eigenvalues can be expressed in polar coordinates as


(14a) $$\begin{array}{l} \lambda_{l,n}=2\pi A{\left(\frac{C{\left(\sigma^{BC}\right)}^{2}}{C\left({\left(\sigma^{AB}\right)}^{2}+{\left(\sigma^{BC}\right)}^{2}\right)+{\left(\sigma^{BC}\right)}^{2}}\right)}^{l+n+1} \end{array}$$

(14b) $$\begin{array}{l} {\text{v}}_{l,n}\left(r,\theta \right)=N_{l,n}r^{l-n}\exp\left(-\frac{r^{2}}{2C}\right){\text{L}}_{n}^{l-n}\left(\frac{r^{2}}{C}\right)\exp i\left(l-n\right)\theta , \end{array}$$

where *N*_*l, n*_ is a normalization factor and ${\text{L}}_{n}^{l-n}$ is an associated Laguerre polynomial. Since $\int_{0}^{\infty }x^{p}e^{-x}{\text{L}}_{q}^{p}{\left(x\right)}^{2}dx=\left(p+q\right)!/q!$, the normalization factor can be derived as

(15) $$\begin{array}{l} N_{l,n}=\{\begin{array}{cc} \sqrt{\frac{2}{\pi C{\left(\sigma^{AB}\right)}^{2}}},\; & l=n\\ \sqrt{\frac{n!}{\pi l!C^{l-n+1}{\left(\sigma^{AB}\right)}^{2}}},\; & \text{otherwise}, \end{array} \end{array}$$

where the factor of 2 difference occurs for the case *l* = *n* because the integral for the angular component is over cos (0θ), a constant.

**Figure 2:**
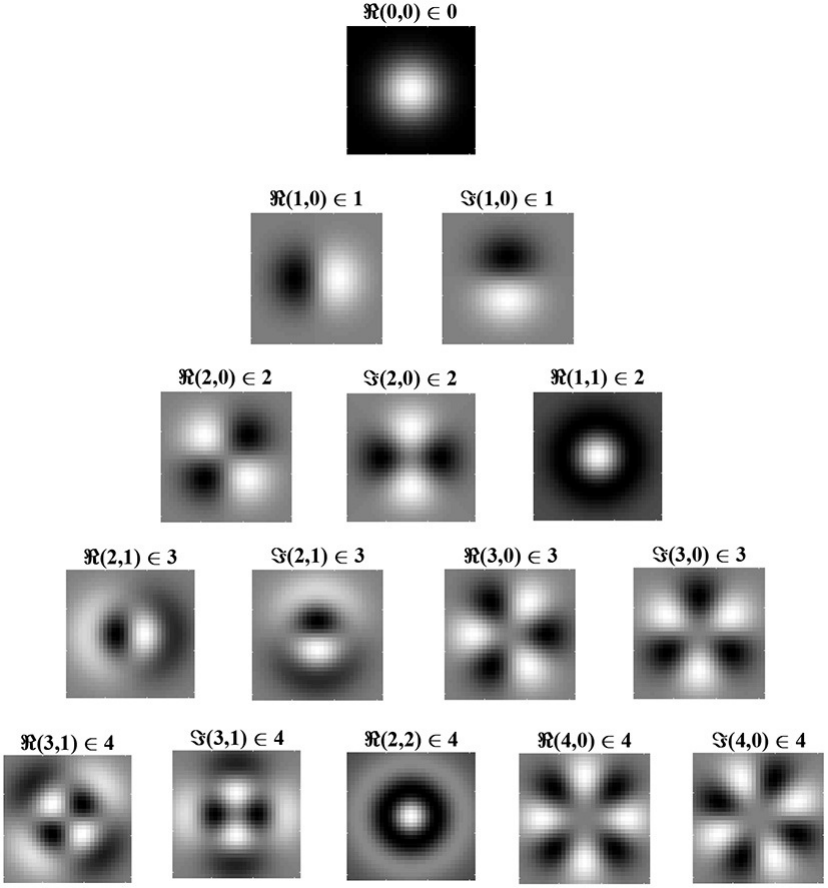
Radial eigenvalues and eigenfunctions of the simplified learning equation, Equation (12), given in Equation (14) for pairs of indices, (*l,n*) ∈ λ_*l,n*_. Eigenvalues are ordered by *l* + *n*, with *l* + *n* = 0 giving the largest eigenvalue and, hence, (0, 0) being the leading eigenfunction. Eigenfunctions in the same row have the same eigenvalue and are, therefore, degenerate. Eigenfunctions are given for both the real part of Equation (14b) (i.e., the cosine angular component) and for the imaginary part, denoted by ℜ and ℑ, respectively. From the figure, it can be seen that, when *l* = *n*, the eigenfunction is radially symmetric, being fully determined by the radial component of the eigenfunction. White indicates positive regions of synaptic weights, while black indicates negative regions. The leading eigenfunction is all positive and dominates learning. As *l* − *n* increases, the frequency of the angular component increases.

Eigenfunctions up to order 4 are shown in Figure 2 in order of decreasing eigenvalue, λ. The eigenfunctions are ordered by *l* + *n*, where *n* controls the shape of the Laguerre polynomial and *l* − *n* controls the angular frequency. The eigenfunction with the largest eigenvalue has order *l* + *n* = 0 and is radially symmetric with all positive synaptic weights. Consequently, for the simplified learning equation described in Equation (12) and after learning for a sufficiently long period, the synaptic weight structure of a layer *C* postsynaptic neuron will be all excitatory connections with weights at the upper bound.

For completeness, we derive the eigenfunctions and eigenvalues of Linsker (1986b) network using Cartesian coordinates, the solution of which is a special case of that found in Wimbauer et al. (1997b). We show that a weighted sum of the Cartesian eigenfunctions produces the radial eigenfunctions, thus establishing equivalence. The derivations are given in Appendix D. For eigenvalues indexed by order *u* and *v*, for the *x* and *y* dimensions respectively, eigenfunction and eigenvalue pairs are given by


(16a) $$\begin{array}{l} \lambda_{u,v}=2\pi {\left(\sigma^{AB}\right)}^{2}q^{u+v+1} \end{array}$$

(16b) $$\begin{array}{l} {\text{v}}_{u,v}\left(\frac{x}{\sqrt{C}},\frac{y}{\sqrt{C}}\right)=\frac{1}{\sqrt{2^{u}u!}}\frac{1}{\sqrt{2^{v}v!}}{\text{H}}_{u}\left(\frac{x}{\sqrt{C}}\right){\text{H}}_{v}\left(\frac{y}{\sqrt{C}}\right)\\ \exp\left(-\frac{x^{2}+y^{2}}{2C}\right). \end{array}$$

**Figure 3:**
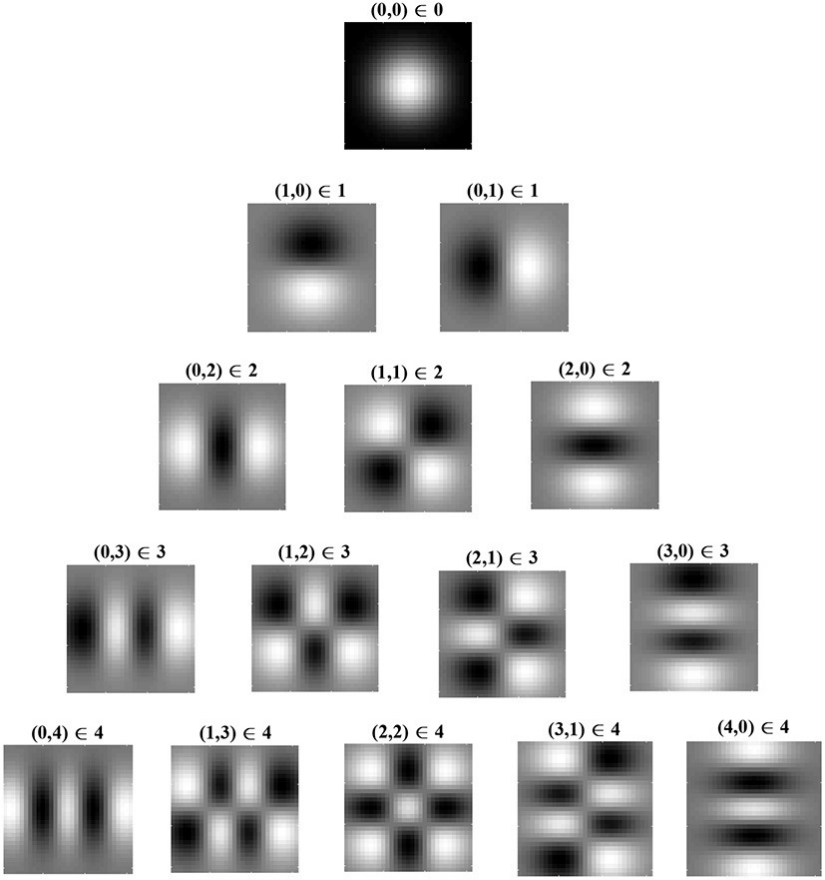
Cartesian eigenfunctions of the simplified learning equation, Equation (61), defined for index pairs (*u, v*). Eigenvalues are determined by *u* + *v*, where (0, 0) has the largest eigenvalue and therefore ${\text{v}}_{0,0}\left(\frac{x}{\sqrt{C}},\frac{y}{\sqrt{C}}\right)$ is the leading eigenfunction, dominating learning. Eigenfunctions in the same row have the same eigenvalue and are, therefore, degenerate. Eigenvalues decrease with descending rows. Regions of white indicate positive synaptic weights, while black indicates negative weights. The leading eigenfunction has all positive synapse weights.

**Figure 4:**
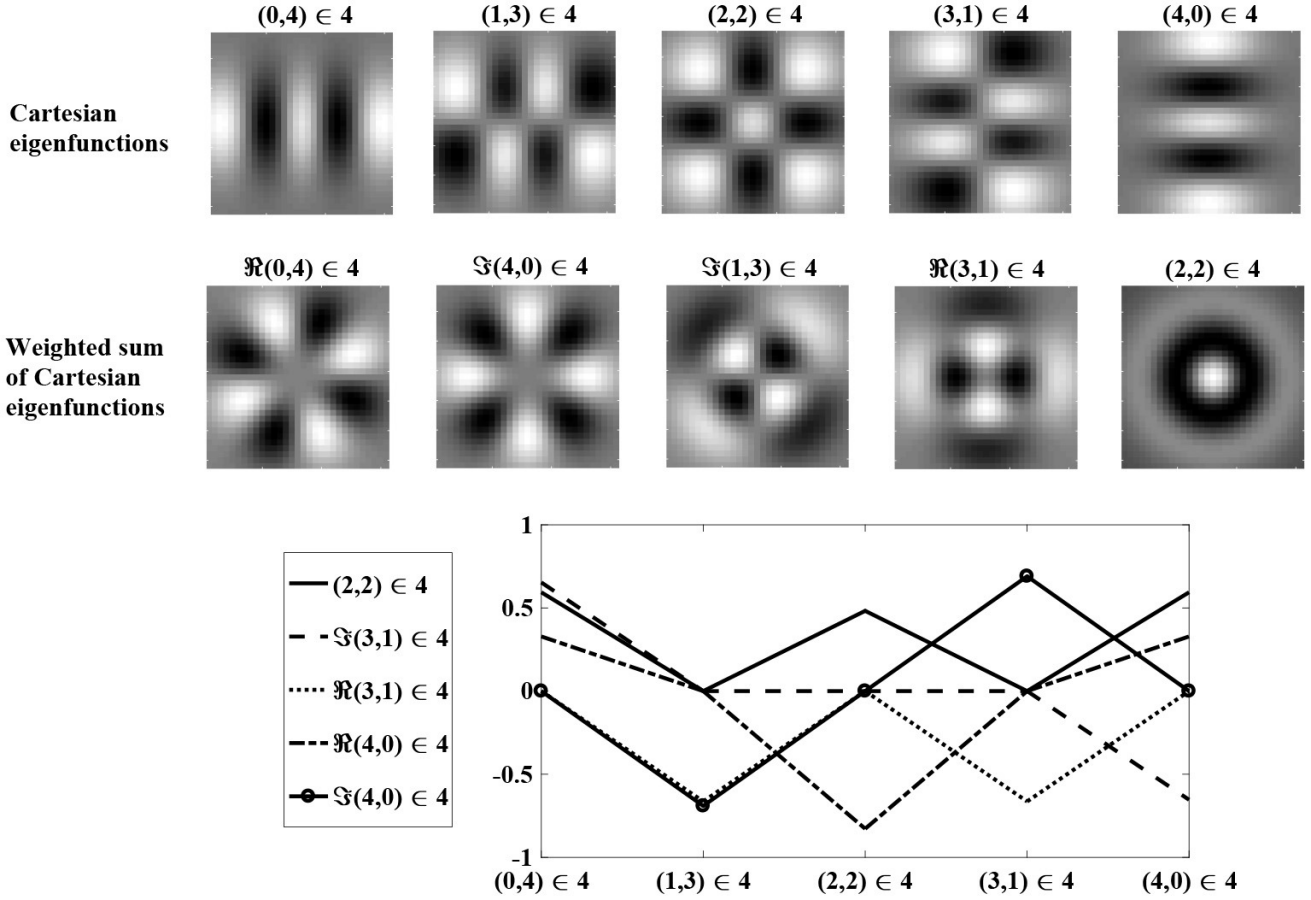
A weighted sum of degenerate Cartesian eigenfunctions of order *u* + *v* = 4 are used to generate radial eigenfunctions of order *l* + *n* = 4. Weights are determined using maximum likelihood regression. The **top** row shows degenerate Cartesian eigenfunctions of order 4. The **middle** row shows the weighted sum of the Cartesian eigenfunctions, reproducing the radial eigenfunctions in the bottom row of Figure 2. The **bottom** row shows the regression weights.

Figure 3 shows Cartesian eigenfunctions up to the fourth order, which is determined by *u* + *v*. The eigenfunctions are shown in order of decreasing eigenvalue, so that the eigenfunction with the largest eigenvalue is of order *u* + *v* = 0. This eigenfunction is radially symmetric, with all positive weights. A radial eigenfunction with a given degenerate order can be reconstructed as a weighted linear sum of Cartesian eigenfunctions of the same order (Figure 4). Consequently, the Cartesian eigenfunctions of the simplified learning equation described in Equation (61) give the same result as the radial eigenfunctions, shown in Figure 2. After sustained learning, the weight structure of a neuron in layer *C* will have all synapses at the upper bound.

### 3.2. Radial eigenfunctions of the full learning equation

While covariance between the activity of layer *B* input neurons primarily drives the structure of the layer *C* cell, the $k_{1}^{BC}$ and $k_{2}^{BC}$ terms control the homeostatic equilibrium. MacKay and Miller (1990) empirically showed that the choice of $k_{2}^{BC}$ can change the structure of the dominant eigenfunction, and hence the resultant receptive field of a layer *C* cell. As Figure 2 shows, for the simplified system, the leading eigenvalue has all synapses at the upper or the lower bound. For a negative value of $k_{2}^{BC}$, homeostasis can only be reached if some of the synapses are negative. To determine the impact of the learning constant, we find an analytical expression for the eigenfunctions of the full learning equation, Equation (11), by conducting a perturbation analysis on the simplified learning equation, in Equation (11).

The full derivation is detailed in Appendix C.2. The eigenfunctions of the first order perturbation are equal to those of the simplified equation, Equation (14). However, the eigenvalues are altered by the addition of the learning constants according to

(17) $$\begin{array}{l} \lambda_{l,n}^{1}=\lambda_{l,n}+W_{l,n}, \end{array}$$

where

(18) $$\begin{array}{l} W_{l,n}=\pi C^{l-n+1}k_{2}^{BC}N_{l,n}^{2}\frac{\Gamma \left(l+n+1\right){\left(\alpha -1\right)}^{2n}}{n{!}^{2}\alpha^{l+n+1}}\\ \;{\;}_{2}F_{1}\left(-n,n;l-n;\frac{\alpha \left(\alpha -2\right)}{{\left(\alpha -1\right)}^{2}}\right), \end{array}$$

and _2_*F*_1_() is the hypergeometric function. As detailed in Appendix C.2, the only non-zero perturbations are where *l* + *n* is even and *l* = *n*, which happens only once for each even order degenerate eigenfunction set.

**Figure 5:**
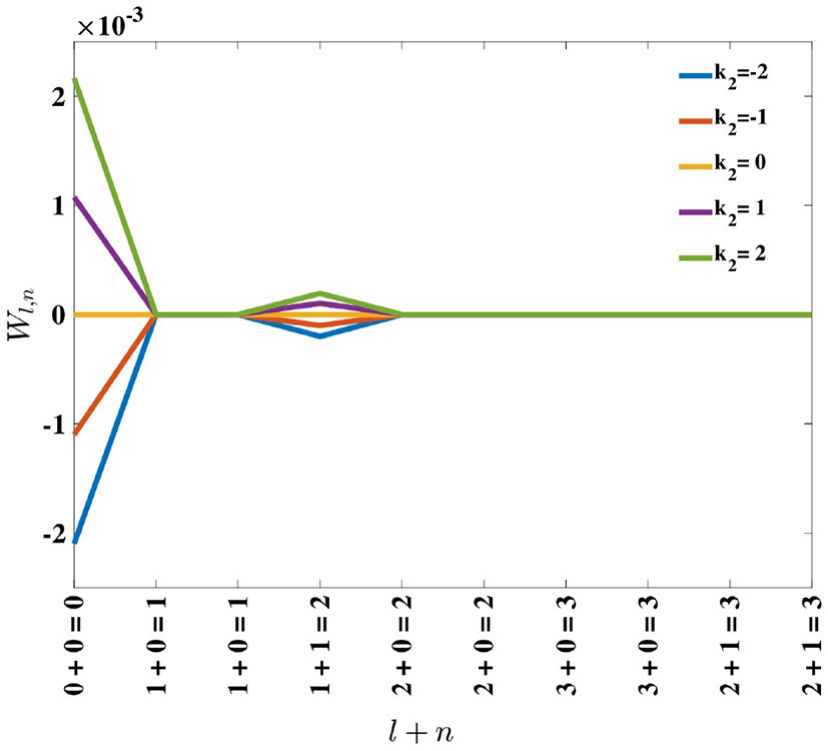
Effect of adding the perturbation term, *k*_2_, on eigenvalues λ_*l,n*_, represented by *W*_*l,n*_ in Equation (17). For positive *k*_2_, the perturbation results in *W*_*l,n*_ being positive, while for negative *k*_2_, the perturbation causes *W*_*l,n*_ to be negative.

Inspection of Equation (18) reveals that, for positive *k*_2_, perturbation of the eigenvalues is positive and monotonically decreasing with *l* + *n*. Consequently, the order of the eigenvalues remains the same. For negative *k*_2_, the perturbation on the eigenvalues is negative and monotonically increasing with eigenfunction order, *l* + *n* (see Figure 5). Since these perturbations are being added to the original eigenvalues, which are positive, the result can be a change in the dominant eigenfunction. This result supports the empirical findings by MacKay and Miller (1990) who showed the emergence of a spatial opponent cell in *C*, where *l* + *n* = 0 for small values of *k*_2_, and bi-lobed neurons with *l* + *n* = 1, for larger values of *k*_2_.

## 4. Emergence of radial orientation selectivity

The original network proposed by Linsker (1986b), and for which we have calculated the eigenfunctions, made an implicit assumption that neurons within each layer were evenly distributed and that receptive fields of all neurons in a layer were statistically identical, drawn from the same synaptic connectivity distribution. However, it is known that some biological neuron layers show an uneven density of cells across the lamina, and contain receptive fields with different statistical properties.

We consider the impact of changing neuron density as a function of distance to the layer center. We assume that a consequence of this is that the radius of a neuron's synaptic connectivity distribution becomes dependent on the neuron's position in the layer. That is, where neurons are spread further apart there is an increase in connectivity radius to compensate. For simplicity assume that a postsynaptic neurons's arbor is within a sufficiently small area that the presynaptic neuron connection density is parameterised by a constant radius. If we denote the spatial center of the neuron layer by *c* and consider this point to have location vector [0, 0], then a postsynaptic cell, located at [*x*_*ic*_, *y*_*ic*_], has connection density that is a function of the magnitude of its position,

(19) $$\begin{array}{l} d_{ic}^{B}={\left(x_{i}^{2}+y_{i}^{2}\right)}^{½}. \end{array}$$

Let the radius of a cell be a linear function of its radial distance to the layer center, such that

(20) $$\begin{array}{l} \sigma_{i}^{AB}=d_{ic}^{B}\sigma^{AB}. \end{array}$$

In this scenario, the probability of presynaptic neuron, *m*, in layer *A*, generating a synaptic connection to postsynaptic neuron, *i*, in layer *B*, is given by

(21) $$\begin{array}{l} pN\left(\left(x_{mi},y_{mi}\right);\frac{1}{2}{\left(\sigma^{AB}\right)}^{2}\right)=\frac{1}{\pi {\left(d_{ic}^{B}\right)}^{2}{\left(\sigma^{AB}\right)}^{2}}\\ \;\exp\left(-\frac{x_{mi}^{2}+y_{mi}^{2}}{{\left(d_{ic}^{B}\right)}^{2}{\left(\sigma^{AB}\right)}^{2}}\right). \end{array}$$

In Appendix E, we calculate the expected number of shared inputs between two neurons in layer *B*. This is important to consider as shared inputs is the source of correlation between layer *B* neurons, which then triggers the emergence of spatial opponent neurons in Linsker (1986b) network. In the case of the synaptic connection radius increasing linearly with distance from the center of the neuron layer, the expected number of shared inputs between two layer *B* neurons is found to be

(22) $$\begin{array}{l} \text{E}\left[N^{AB};\left[x_{i},y_{i}\right],\left[x_{j},y_{j}\right]\right]=\frac{{\left(N^{AB}\right)}^{2}}{\pi {\left(\sigma^{AB}\right)}^{2}\left(d_{i}^{2}+d_{j}^{2}\right)}\\ \exp\left(-\frac{d_{ij}^{2}}{{\left(\sigma^{AB}\right)}^{2}\left(d_{i}^{2}+d_{j}^{2}\right)}\right). \end{array}$$

**Figure 6:**
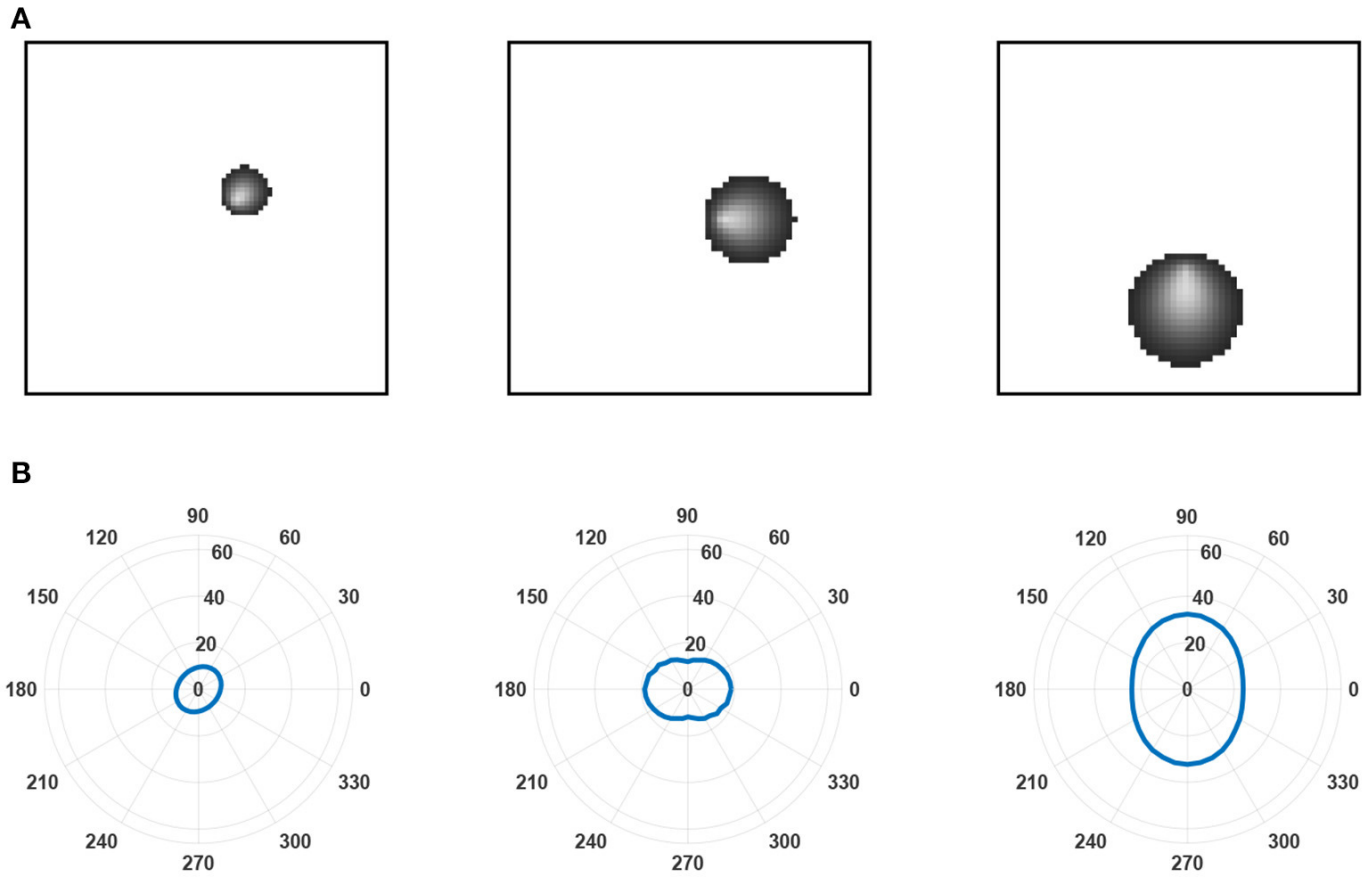
Examples of receptive fields and tuning curves. **(A)** Receptive fields predicted by the leading eigenfunction of three layer *C* neurons at different locations relative to the center of layer *B*. The layer is small relative to the receptive field size of the layer *C* neurons to highlight changes in receptive field size and the radial orientation selectivity of selected layer *C* neurons. These features emerge from a linearly increasing arbor radius in synaptic connectivity between layer *A* and layer *B* neurons. As established by Linsker (1986b), it is the overlap between the arbors of layer *B* neurons that prompts correlation in their activity despite only inputting unstructured noise into layer *A* and, subsequently, generates the structured receptive fields found in the layer *C* neurons. **(B)** Tuning curves generated for each of the layer *C* neurons in **(A)**. Tuning curves were calculated for the spatial frequency prompting the largest response in the neuron.

We simulated this learning equation and plotted the receptive fields of three layer *C* neurons in three different positions relative to a small layer of *B* neurons (see Figure 6). The neurons developed radial orientation tuning, with tuning curves showing an orientation directed toward the center of the lamina.

## 5. Discussion

In this paper, we provide a general expression for the complete set of eigenfunctions for the three-layer feed-forward network proposed by Linsker (1986b). Initially, the homeostatic parameters were set to zero to simplify the learning equation. This result was then extended via a perturbation analysis to provide the complete set of eigenfunctions for the network with non-zero homeostatic parameters.

Linsker (1986b) analysis was integral in revealing how neural learning occurred before the onset of structured environmental input, empirically demonstrating the emergence of spatial opponent neurons in the third layer. MacKay and Miller (1990) provided a stability analysis of Linsker (1986b) network, noting the first six eigenfunctions, determined via an ansatz based on the results of Tang (1990). MacKay and Miller (1990) showed that the receptive field structure of cells in the third layer could be either spatial opponent neuron or bi-lobed neurons, depending upon the value of the homeostatic parameters. Similarly, Walton and Bissest (1992) extended Linsker (1986b) network to the auditory system, considering the morphology of the resulting neuron based on the homeostatic parameters of the system. In this paper, we provide the complete set of eigenvalues for the full learning equation, enabling an exact calculation of the homeostatic parameters required to induce this change, and a quantitative analysis on the parameter space.

Linsker (1986a) showed that augmenting the network with additional layers prompts the development of orientation selective neurons. However, given the absence of a complete mathematical framework for the three-layer network, there has been limited analysis provided for the development of orientation selective neurons in Linsker (1986a) network (MacKay and Miller, 1990; Miller, 1990; Wimbauer et al., 1998). Yamakazi (2002) provided an analysis of deeper layers, essentially based on an ansatz for the eigenfunctions for the three-layer network. The results in this paper provide the foundation for analysis of larger networks and hence the development of features other than spatial opponent neurons. As the system is radially symmetric in connectivity distribution, radial eigenfunctions provide a natural coordinate system that will facilitate future work on more complex network and parameter regimes.

The results of this study demonstrate that relaxing the assumption of evenly distributed neurons across the layer can change the receptive fields that emerge in the third layer. Similar to the distribution in the retina, we examined a decrease in neuron density with increasing distance from the center of the layer and, consequently, an increase in synaptic connectivity radius. We analytically derived an expression for the learning equation in the third layer, as a result of a radially dependent connectivity distribution between the first and second layers. The eigenfunctions for the learning equation were empirically calculated, showing that orientation selective neurons emerge. Interestingly, the preferred orientation of the neurons was the radial orientation toward the center of the laminar. The radial bias is more pronounced for peripheral neurons, which accords with experimental results (Freeman et al., 2011).

It is well established that neural density changes as a function of eccentricity in the retina (Sjöstrand et al., 1999; Watson, 2014), and receptive field sizes of neurons in the primary visual cortex increase with stimulus eccentricity (Smith et al., 2001; Wurbs et al., 2013). Furthermore, it is known that orientation selectivity in the primary visual cortex is biased toward radial orientation, in that an orientation selective neuron in the primary visual cortex is more likely to be oriented toward the center of the retina (Rodionova et al., 2004; Sasaki et al., 2006; Antinucci and Hindges, 2018). However, the origin of radial orientation selectivity has not yet been confirmed, and hence its emergence from inhomogeneous cell density has significance as a potential mechanism. It should be noted that experimentally measured orientation bias has been shown to emerge as an artifact of visual gratings (Scholl et al., 2013). However, given that radial orientation bias has been established using a range of stimuli, such as small bars (Philips and Chakravarthy, 2017), and random dots in conjunction with reverse correlation (Mareschal et al., 2006), its presence is now well established.

## Data availability statement

The original contributions presented in the study are included in the article/Supplementary material, further inquiries can be directed to the corresponding author.

## Author contributions

CD wrote the code and developed the idea under the supervision of AB and DG. All authors contributed to the article and approved the submitted version.
