# Supplementary material for: Emergence of radial orientation selectivity: Effect of cell density changes and eccentricity in a layered network
Source: Front Comput Neurosci. 2022 Dec 13;16:881046. doi: 10.3389/fncom.2022.881046 (PMC9793711; doi:10.3389/fncom.2022.881046)
Supplement: Supplementary file 1 [file Data_Sheet_1.pdf]

Appendix

A Expected number of shared inputs

To examine network dynamics, it is necessary to ascertain the expected number of shared connections between two neurons. The number of shared connections from a presynaptic layer to two neurons in the postsynaptic layer, say i and j, depends on the radial distance between them since the synaptic connection density for each is a Gaussian function of distance (see Fig. 1). We assume for simplicity and without loss of generality that i and j differ only in their x coordinate so that $d_{ij}^{B} = x_{mi} - x_{mj}$.

Center the Cartesian coordinates describing a neuron's position in the laminar on one of the postsynaptic neurons, say i, so that the other postsynaptic neuron, say j, lies on the x axis. From Eq. (1), neuron, m, in layer A, has a probability of connecting to neuron i in layer B of $p_N\left(x_{mi}, y_{mi}\,;\mathbf{0},\Sigma^A\right)$ and a probability of connecting to neuron j in layer B of $p_N\left(x_{mj}, y_{mj}\,;\mathbf{0},\Sigma^A\right) = p_N\left(x_{mi} - d_{ij}^{B}, y_{mi}\,;\mathbf{0},\Sigma^A\right)$. The probability of the presynaptic neuron connecting to both postsynaptic neurons i and j is simply the product of the probability of each individual connection being made. The expected number of common connections can be determined by summing this joint probability over the layer or, in the continuous limit, integrating the joint probability over the layer of presynaptic neurons. If N^{AB} denotes the number of synaptic connections from layer A to a layer B neuron and $N^{BB}(d)$ the number of shared connections between two postsynaptic neurons in layer B separated by a distance of d, then in the continuous limit,

$$N^{BB}(d) = (N^{AB})^2 \iint\limits_{xy} p_N\left(x,y\,;\mathbf{0},\Sigma^A\right) p_N\left(x-d,y\,;\mathbf{0},\Sigma^A\right) dx\,dy, \tag{23}$$

where the sub- and super-scripts on distance parameters have been dropped to aid readability. This can be expanded as

$$\begin{aligned}
N^{BB}(d) &= (N^{AB})^2 \iint\limits_{xy} \frac{1}{(\pi(\sigma^{AB})^2)^2} \exp\left(-\frac{x^2+y^2}{(\sigma^{AB})^2}\right) \exp\left(-\frac{(x-d)^2+y^2}{(\sigma^{AB})^2}\right) dx\,dy \\
&= \frac{(N^{AB})^2}{(\pi(\sigma^{AB})^2)^2} \iint\limits_{xy} \exp\left(-\frac{2x^2+2y^2+d^2-2xd}{(\sigma^{AB})^2}\right) dx\,dy \\
&= \frac{(N^{AB})^2}{(\pi(\sigma^{AB})^2)^2} \iint\limits_{xy} \exp\left(-\frac{2\left(\left(x-\frac{d}{2}\right)^2+y^2+\frac{d^2}{4}\right)}{(\sigma^{AB})^2}\right) dx\,dy.
\end{aligned} \tag{24}$$

Introduce $x' = x - {}^{d}/_{2}$, so that

$$\begin{aligned}
N^{BB}(d) &= \exp\left(-\frac{d^2}{2(\sigma^{AB})^2}\right) \frac{(N^{AB})^2}{(\pi(\sigma^{AB})^2)^2} \iint\limits_{xy} \exp\left(-\frac{2\left(x'^2+y^2\right)}{(\sigma^{AB})^2}\right) dx\,dy \\
&= \exp\left(-\frac{d^2}{2(\sigma^{AB})^2}\right) \frac{(N^{AB})^2}{(\pi(\sigma^{AB})^2)^2} \sqrt{\frac{\pi(\sigma^{AB})^2}{2}} \sqrt{\frac{\pi(\sigma^{AB})^2}{2}} \\
&= \frac{(N^{AB})^2}{2\pi(\sigma^{AB})^2} \exp\left(-\frac{d^2}{2(\sigma^{AB})^2}\right),
\end{aligned} \tag{25}$$

using the identity $\int_{-\infty}^{\infty} \exp\left(-ax^2\right) = \sqrt{\pi/a}$.

This result demonstrates that the number of shared connections between two neurons with Gaussian synaptic connection densities is itself a Gaussian function of the radial distance between the neurons with a variance that is half the value of the synaptic connection density radius. This means that a postsynaptic neuron is expected to have the most common connections with itself, for which $d = 0$. Additionally, for small variance or connection radius, a postsynaptic neuron will share many connections with proximate neighbors, with the number of shared connections falling off quickly with distance. Since the expected number of synaptic inputs is constant, a large connection radius implies that the neuron will have shared connections with neurons comparatively distal to it, since nearby neurons will have comparatively fewer shared connections.

B Covariance of neural activity in layer B

Expressions for the covariance of layer B neurons are derived here. Sample covariance between two postsynaptic neuron rates in layer B, say f_i^B and f_j^B, for neurons i and j, respectively, is calculated as

$$\operatorname{cov}\left(f_i^B, f_j^B\right) = \mathrm{E}\left[f_i^B f_j^B\right] - \mathrm{E}\left[f_i^B\right]\mathrm{E}\left[f_j^B\right]. \tag{26}$$

For unitary weights from layer A to layer B, Eq. (4a), can be employed to give

$$\begin{aligned}
\operatorname{cov}\left(f_i^B, f_j^B\right) &= \mathrm{E}\left[\left(R_a^B + \sum_m f_m^A\right)\left(R_a^B + \sum_n f_n^A\right)\right] - \mathrm{E}\left[R_a^B + \sum_m f_m^A\right]\mathrm{E}\left[R_a^B + \sum_n f_n^A\right] \\
&= (R_a^B)^2 + 2R_a^B N^{AB}\overline{f^A} + \mathrm{E}\left[\sum_m \sum_n f_m^A f_n^A\right] - \left((R_a^B)^2 + 2R_a^B N^{AB}\overline{f^A} + (N^{AB}\overline{f^A})^2\right) \\
&= \left(\mathrm{E}\left[\sum_m \sum_n f_m^A f_n^A\right] - (N^{AB}\overline{f^A})^2\right).
\end{aligned} \tag{27}$$

Layer A neurons are uncorrelated so that the only non-zero contribution to this sum occurs when a layer A neuron has a synaptic connection to each of the layer B neurons under consideration. In this case the input rates are fully correlated, so that the contribution to covariance is proportional to the layer A firing rate.

C Derivation of radial eigenfunctions

C.1 Simplified learning equation

We start by decomposing the weight functions, $w(r,\theta)$, of Eq. (12), into a sum of independent components that are dense in the space using a Fourier series. Therefore, expressing the exponential in its infinite series form, we get

$$\begin{aligned}
\eta \sum_{l=0}^{\infty} \lambda_l \left(g_l(r)\cos(l\theta) + \tilde{g}_l(r)\sin(l\theta)\right) = A(\sigma^{AB})^2 \exp\left(-\frac{r^2}{2}\left(\frac{2(\sigma^{AB})^2 + (\sigma^{BC})^2}{(\sigma^{BC})^2}\right)\right) \int_0^{\infty} d\tilde{r}\,\tilde{r} \exp\left(-\frac{\tilde{r}^2}{2}\left(\frac{(\sigma^{AB})^2 + (\sigma^{BC})^2}{(\sigma^{BC})^2}\right)\right) \\
\int_0^{2\pi} d\tilde{\theta} \sum_{n=0}^{\infty} \left(r\tilde{r}\cos\left(\theta - \tilde{\theta}\right)\right)^n \frac{1}{n!} \sum_{l'=0}^{\infty} \left(g_{l'}(\tilde{r})\cos\left(l'\tilde{\theta}\right) + \tilde{g}_{l'}(\tilde{r})\sin\left(l'\tilde{\theta}\right)\right).
\end{aligned} \tag{28}$$

Rearrange the sum and integral terms to give

$$\begin{aligned}
\eta \sum_{l=0}^{\infty} \lambda_l \left(g_l(r)\cos(l\theta) + \tilde{g}_l(r)\sin(l\theta)\right) = A(\sigma^{AB})^2 \exp\left(-\frac{r^2}{2}\left(\frac{2(\sigma^{AB})^2 + (\sigma^{BC})^2}{(\sigma^{BC})^2}\right)\right) \int_0^{\infty} d\tilde{r}\,\tilde{r} \exp\left(-\frac{\tilde{r}^2}{2}\left(\frac{2(\sigma^{AB})^2 + (\sigma^{BC})^2}{(\sigma^{BC})^2}\right)\right) \\
\sum_{l'=0}^{\infty} \sum_{n=0}^{\infty} \frac{1}{n!} (r\tilde{r})^n \int_0^{2\pi} d\tilde{\theta} \cos^n\left(\theta - \tilde{\theta}\right) \left(g_{l'}(\tilde{r})\cos\left(l'\tilde{\theta}\right) + \tilde{g}_{l'}(\tilde{r})\sin\left(l'\tilde{\theta}\right)\right).
\end{aligned} \tag{29}$$

Now we consider just the inner integral over $\tilde{\theta}$,

$$\int_0^{2\pi} d\tilde{\theta} \left(\cos^n\left(\theta - \tilde{\theta}\right)\right) \left(g_{l'}(\tilde{r})\cos\left(l'\tilde{\theta}\right) + \tilde{g}_{l'}(\tilde{r})\sin\left(l'\tilde{\theta}\right)\right). \tag{30}$$

A general expression for $\left(\cos^n(\phi)\right)$ can be found by writing it as

$$
\begin{aligned}
\cos^n(\phi) =& \frac{1}{2^n}\left(e^{-\mathrm{i}\phi}+e^{\mathrm{i}\phi}\right)^n \\
=& \frac{1}{2^n}\sum_{k=0}^{n}\binom{n}{k}\left(e^{-\mathrm{i}\phi}\right)^k\left(e^{\mathrm{i}\phi}\right)^{n-k} = \frac{1}{2^n}\sum_{k=0}^{n}\binom{n}{k}\left(e^{-\mathrm{i}\phi}\right)^{n-k}\left(e^{\mathrm{i}\phi}\right)^k \\
=& \frac{1}{2^n}\sum_{k=0}^{n}\binom{n}{k}\left(e^{-\mathrm{i}\phi(n-2k)}\right) = \frac{1}{2^n}\sum_{k=0}^{n}\binom{n}{k}\left(+e^{\mathrm{i}\phi(n-2k)}\right) \\
=& \frac{1}{2^{n+1}}\sum_{k=0}^{n}\binom{n}{k}\left(e^{-\mathrm{i}\phi(n-2k)}+e^{\mathrm{i}\phi(n-2k)}\right) \\
=& \frac{1}{2^n}\sum_{k=0}^{n}\binom{n}{k}\cos\left(\phi(n-2k)\right) \\
=& \begin{cases} \frac{1}{2^{n-1}}\sum\limits_{k=0}^{\frac{n-1}{2}}\binom{n}{k}\cos\left(\phi(n-2k)\right), & \text{for } n \text{ odd} \\ \frac{1}{2^n}\binom{n}{\frac{n}{2}}+\frac{1}{2^{n-1}}\sum\limits_{k=0}^{\frac{n}{2}-1}\binom{n}{k}\cos\left(\phi(n-2k)\right), & \text{for } n \text{ even} \end{cases}
\end{aligned}
\tag{31}
$$

Application of this result to Eq. (30) gives

$$
\begin{aligned}
&\int_0^{2\pi} d\tilde{\theta}\cos^n\left(\theta-\tilde{\theta}\right)\left(g_{l'}(\tilde{r})\cos\left(l'\tilde{\theta}\right)+\tilde{g}_{l'}(\tilde{r})\sin\left(l'\tilde{\theta}\right)\right) \\
&\quad= \frac{1}{2^n}\int_0^{2\pi} d\tilde{\theta}\sum_{k=0}^{n}\binom{n}{k}\cos\left((\theta-\tilde{\theta})(n-2k)\right)\left(g_{l'}(\tilde{r})\cos\left(l'\tilde{\theta}\right)+\tilde{g}_{l'}(\tilde{r})\sin\left(l'\tilde{\theta}\right)\right) \\
&\quad= \frac{1}{2^n}\sum_{k=0}^{n}\binom{n}{k}\int_0^{2\pi} d\tilde{\theta}\cos\left((\theta-\tilde{\theta})(n-2k)\right)g_{l'}(\tilde{r})\cos\left(l'\tilde{\theta}\right) \\
&\qquad+\frac{1}{2^n}\sum_{k=0}^{n}\binom{n}{k}\int_0^{2\pi} d\tilde{\theta}\cos\left((\theta-\tilde{\theta})(n-2k)\right)\tilde{g}_{l'}(\tilde{r})\sin\left(l'\tilde{\theta}\right) \\
&\quad= \frac{1}{2^{n+1}}\sum_{k=0}^{n}\binom{n}{k}\int_0^{2\pi} d\tilde{\theta}g_{l'}(\tilde{r})\left(\cos\left((\theta-\tilde{\theta})(n-2k)+l'\tilde{\theta}\right)+\cos\left(-(\theta-\tilde{\theta})(n-2k)+l'\tilde{\theta}\right)\right) \\
&\qquad+\frac{1}{2^{n+1}}\sum_{k=0}^{n}\binom{n}{k}\int_0^{2\pi} d\tilde{\theta}\tilde{g}_{l'}(\tilde{r})\left(\sin\left((\theta-\tilde{\theta})(n-2k)+l'\tilde{\theta}\right)-\sin\left(-(\theta-\tilde{\theta})(n-2k)+l'\tilde{\theta}\right)\right) \\
&\quad= \frac{1}{2^{n+1}}\sum_{k=0}^{n}\binom{n}{k}\int_0^{2\pi} d\tilde{\theta}g_{l'}(\tilde{r})\left(\cos\left(\theta(n-2k)+\tilde{\theta}(l'-(n-2k))\right)+\cos\left(\theta(n-2k)+\tilde{\theta}(l'+(n-2k))\right)\right) \\
&\qquad+\frac{1}{2^{n+1}}\sum_{k=0}^{n}\binom{n}{k}\int_0^{2\pi} d\tilde{\theta}\tilde{g}_{l'}(\tilde{r})\left(\sin\left(\theta(n-2k)+\tilde{\theta}(l'-(n-2k))\right)-\sin\left(\theta(n-2k)+\tilde{\theta}(l'+(n-2k))\right)\right) \\
&\quad= \begin{cases}
\frac{1}{2^n}\sum\limits_{k=0}^{\frac{n-1}{2}}\binom{n}{k}\int_0^{2\pi} d\tilde{\theta}\left[g_{l'}(\tilde{r})\left(\cos\left(\theta(n-2k)+\tilde{\theta}(l'-(n-2k))\right)+\cos\left(\theta(n-2k)+\tilde{\theta}(l'+(n-2k))\right)\right)\right. \\
\quad\left.+\,\tilde{g}_{l'}(\tilde{r})\left(\sin\left(\theta(n-2k)+\tilde{\theta}(l'-(n-2k))\right)+\sin\left(\theta(n-2k)-\tilde{\theta}(l'+(n-2k))\right)\right)\right], & \text{for } n \text{ odd} \\
\frac{1}{2^{n+1}}\binom{n}{\frac{n}{2}}\int_0^{2\pi} d\tilde{\theta}\left(\cos\left(l'\tilde{\theta}\right)+\sin\left(l'\tilde{\theta}\right)\right) \\
\quad+\frac{1}{2^n}\sum\limits_{k=0}^{\frac{n}{2}-1}\binom{n}{k}\int_0^{2\pi} d\tilde{\theta}\left[g_{l'}(\tilde{r})\left(\cos\left(\theta(n-2k)+\tilde{\theta}(l'-(n-2k))\right)+\cos\left(\theta(n-2k)+\tilde{\theta}(l'+(n-2k))\right)\right)\right. \\
\quad\left.+\tilde{g}_{l'}(\tilde{r})\left(\sin\left(\theta(n-2k)+\tilde{\theta}(l'-(n-2k))\right)+\sin\left(\theta(n-2k)-\tilde{\theta}(l'+(n-2k))\right)\right)\right], & \text{for } n \text{ even}
\end{cases}
\end{aligned}
\tag{32}
$$

All of the integrals within the binomial sum term will evaluate to zero since the functions are periodic in 2π and centred around a mean of zero, except those for which $l' = n-2k$, because in these cases the $\tilde{\theta}$ terms cancel, and therefore the integration is over a constant. For odd l', this can only happen for odd n, and for even l', this can only happen for even n, when $k = \frac{n-l'}{2}$

such that $0 \le k \le \frac{n-1}{2}$. Consequently, the sinusoidal term for which the $\tilde{\theta}$ coefficient is $l' + n - 2k$ will always integrate to 0, since $l' + 2k - n \ge 0$ for all k. The additional term for even n will only be non-zero when l' is 0. Thus equation Eq. (32) evaluates to

$$\int_0^{2\pi} d\tilde{\theta} \cos^n\left(\theta - \tilde{\theta}\right)\left(g_{l'}\left(\tilde{r}\right)\cos\left(l'\tilde{\theta}\right) + \tilde{g}_{l'}\left(\tilde{r}\right)\sin\left(l'\tilde{\theta}\right)\right)$$
$$= \begin{cases} \frac{2\pi}{2^{n+1}}\binom{n}{\frac{n}{2}} g_{l'}\left(\tilde{r}\right), & \text{for } l' = 0 \text{ and } n \text{ even} \\ \frac{2\pi}{2^n}\binom{n}{\frac{n-l'}{2}} g_{l'}\left(\tilde{r}\right)\cos\left(l'\theta\right) + \frac{2\pi}{2^n}\binom{n}{\frac{n-l'}{2}}\tilde{g}_{l'}\left(\tilde{r}\right)\sin\left(l'\theta\right), & \text{for } 0 \le \frac{n-l'}{2} \le \frac{n-1}{2} \text{ an integer} \\ 0, & \text{otherwise.} \end{cases} \tag{33}$$

Note that for $n - l' \ge 0$ we require $n \ge l'$, and thus the infinite sum over l' in Eq. (29) can be truncated.

Incorporating this result into the infinite sums from Eq. (29), separating the odd and even terms for n and l', and focusing only on the cosine components for the interim, gives,

$$\begin{aligned} &\sum_{l'=0}^{\infty}\sum_{n=0}^{\infty}\int_0^{2\pi} d\tilde{\theta}\cos^n\left(\theta - \tilde{\theta}\right) g_{l'}\left(\tilde{r}\right)\cos\left(l'\tilde{\theta}\right) \\ &\quad = \sum_{n=0}^{\infty}\sum_{l'=0}^{n}\left[\frac{2\pi}{2^{2n}}\binom{2n}{n-l'} g_{2l'}\left(\tilde{r}\right)\cos\left(2l'\theta\right) + \frac{2\pi}{2^{2n+1}}\binom{2n+1}{n-l'} g_{2l'+1}\left(\tilde{r}\right)\cos\left((2l'+1)\theta\right)\right] \\ &\quad = \sum_{l'=0}^{\infty}\sum_{n=l'}^{\infty}\left[\frac{2\pi}{2^{2n}}\binom{2n}{n-l'} g_{2l'}\left(\tilde{r}\right)\cos\left(2l'\theta\right) + \frac{2\pi}{2^{2n+1}}\binom{2n+1}{n-l'} g_{2l'+1}\left(\tilde{r}\right)\cos\left((2l'+1)\theta\right)\right]. \end{aligned} \tag{34}$$

Let $n' = n - l'$, so that

$$\begin{aligned} &\sum_{l'=0}^{\infty}\sum_{n=0}^{\infty}\int_0^{2\pi} d\tilde{\theta}\cos^n\left(\theta - \tilde{\theta}\right) g_{l'}\left(\tilde{r}\right)\cos\left(l'\tilde{\theta}\right) \\ &\quad = \sum_{l'=0}^{\infty}\sum_{n'=0}^{\infty}\left[\frac{2\pi}{2^{2(n'+l')}}\binom{2(n'+l')}{n'} g_{2l'}\left(\tilde{r}\right)\cos\left(2l'\theta\right) + \frac{2\pi}{2^{2(n'+l')+1}}\binom{2(n'+l')+1}{n'} g_{2l'+1}\left(\tilde{r}\right)\cos\left((2l'+1)\theta\right)\right]. \end{aligned} \tag{35}$$

Note that n' and l' can now be set independently so that the odd and even terms for l' can now be recombined.

$$\sum_{l'=0}^{\infty}\sum_{n'=0}^{\infty}\int_0^{2\pi} d\tilde{\theta}\cos^n\left(\theta - \tilde{\theta}\right) g_{l'}\left(\tilde{r}\right)\cos\left(l'\tilde{\theta}\right) = \sum_{l'=0}^{\infty}\sum_{n'=0}^{\infty}\frac{2\pi}{2^{2n'+l'}}\binom{2n'+l'}{n'} g_{l'}\left(\tilde{r}\right)\cos\left(l'\theta\right). \tag{36}$$

Incorporating this sum into the full equation for Eq. (29), gives,

$$
\begin{aligned}
&\eta \sum_{l=0}^{\infty} \lambda_l \left(g_l\left(r\right)\cos\left(l\theta\right)\ + \tilde{g}_l\left(r\right)\sin\left(l\theta\right)\right) \\
&\quad = A(\sigma^{AB})^2 \exp\left(-\frac{r^2}{2}\left(\frac{2(\sigma^{AB})^2+(\sigma^{BC})^2}{(\sigma^{BC})^2}\right)\right)\int_0^{\infty} d\tilde{r}\,\tilde{r}\exp\left(-\frac{\tilde{r}^2}{2}\left(\frac{2(\sigma^{AB})^2+(\sigma^{BC})^2}{(\sigma^{BC})^2}\right)\right) \\
&\quad \sum_{l'=0}^{\infty}\sum_{n'=0}^{\infty}\binom{2n'+l'}{n'}\frac{1}{(2n'+l')!}\left(r\tilde{r}\right)^{2n'+l'}\frac{2\pi}{2^{2n'+l'}}\left(g_{l'}\left(\tilde{r}\right)\cos\left(l'\theta\right)+\tilde{g}_{l'}\left(\tilde{r}\right)\sin\left(l'\theta\right)\right) \\
&\quad = A(\sigma^{AB})^2 \exp\left(-\frac{r^2}{2}\left(\frac{2(\sigma^{AB})^2+(\sigma^{BC})^2}{(\sigma^{BC})^2}\right)\right)\int_0^{\infty} d\tilde{r}\tilde{r}\exp\left(-\frac{\tilde{r}^2}{2}\left(\frac{2(\sigma^{AB})^2+(\sigma^{BC})^2}{(\sigma^{BC})^2}\right)\right) \\
&\quad \sum_{l'=0}^{\infty}\sum_{n'=0}^{\infty}\frac{2\pi}{n'!(n'+l')!}\left(\frac{r\tilde{r}}{2}\right)^{2n'+l'}\left(g_{l'}\left(\tilde{r}\right)\cos\left(l'\theta\right)+\tilde{g}_{l'}\left(\tilde{r}\right)\sin\left(l'\theta\right)\right) \\
&\quad = 2\pi A(\sigma^{AB})^2 \exp\left(-\frac{r^2}{2}\left(\frac{2(\sigma^{AB})^2+(\sigma^{BC})^2}{(\sigma^{BC})^2}\right)\right)\int_0^{\infty} d\tilde{r}\tilde{r}\exp\left(-\frac{\tilde{r}^2}{2}\left(\frac{2(\sigma^{AB})^2+(\sigma^{BC})^2}{(\sigma^{BC})^2}\right)\right) \\
&\quad \sum_{l'=0}^{\infty}\sum_{n'=0}^{\infty}\frac{1}{n'!\Gamma(n'+l'+1)}\left(\frac{r\tilde{r}}{2}\right)^{2n'+l'}\left(g_{l'}\left(\tilde{r}\right)\cos\left(l'\theta\right)+\tilde{g}_{l'}\left(\tilde{r}\right)\sin\left(l'\theta\right)\right) \\
&\quad = 2\pi A(\sigma^{AB})^2 \exp\left(-\frac{r^2}{2}\left(\frac{2(\sigma^{AB})^2+(\sigma^{BC})^2}{(\sigma^{BC})^2}\right)\right) \\
&\quad \sum_{l'=0}^{\infty}\int_0^{\infty} d\tilde{r}\tilde{r}\exp\left(-\frac{\tilde{r}^2}{2}\left(\frac{2(\sigma^{AB})^2+(\sigma^{BC})^2}{(\sigma^{BC})^2}\right)\right) I_{l'}\left(r\tilde{r}\right)\left(g_{l'}\left(\tilde{r}\right)\cos\left(l'\theta\right)+\tilde{g}_{l'}\left(\tilde{r}\right)\sin\left(l'\theta\right)\right), \quad (37)
\end{aligned}
$$

where $I_{\alpha}\left(x\right)$ is a modified Bessel function of the first kind, of order α, such that

$$
I_{\alpha}\left(x\right) = \sum_{m=0}^{\infty}\frac{1}{m!\Gamma(m+\alpha+1)}\left(\frac{x}{2}\right)^{2m+\alpha}. \quad (38)
$$

We can consider each component in the sum separately, such that

$$
\begin{aligned}
&\eta\lambda_l\left(g_l\left(r\right)\cos\left(l\theta\right)+\tilde{g}_l\left(r\right)\sin\left(l\theta\right)\right) \\
&= 2\pi A(\sigma^{AB})^2 \exp\left(-\frac{r^2}{2}\left(\frac{2(\sigma^{AB})^2+(\sigma^{BC})^2}{(\sigma^{BC})^2}\right)\right)\int_0^{\infty} d\tilde{r}\tilde{r}\exp\left(-\frac{\tilde{r}^2}{2}\left(\frac{2(\sigma^{AB})^2+(\sigma^{BC})^2}{(\sigma^{BC})^2}\right)\right) I_{l'}\left(r\tilde{r}\right)\left(g_{l'}\left(\tilde{r}\right)\cos\left(l'\theta\right)+\tilde{g}_{l'}\left(\tilde{r}\right)\sin\left(l'\theta\right)\right). \quad (39)
\end{aligned}
$$

To derive the eigenfunctions that satisfy Eq. (39), we require weight functions with an exponential of the same form, and polynomials in r that will be of the same order after evaluating the integral. Furthermore, it is well known that Laguerre polynomials are orthogonal over the interval $[0,\infty)$, with respect to the weight function $x^a\exp\left(-x\right)$. Consequently, we propose eigenfunctions of the form

$$
g_{l,n}\left(\tilde{r}\right)\begin{cases}\cos\left((l'-n)\theta\right)\\ \sin\left((l'-n)\theta\right)\end{cases} = N_{l,n}\tilde{r}^{l-n}\exp\left(-\frac{\tilde{r}^2}{2C}\right)\mathrm{L}_n^{l-n}\left(\frac{\tilde{r}^2}{C}\right)\begin{cases}\cos\left((l'-n)\theta\right)\\ \sin\left((l'-n)\theta\right)\end{cases}, \quad (40)
$$

where the additional index, n, denotes the index into eigenvalues of the same order, l, since the solutions are degenerate, and $N_{l,n}$ is a normalisation factor. L_n^{l-n} is an associated Laguerre polynomial. Since $\int_0^{\infty} x^p e^{-x}\mathrm{L}_q^p(x)^2\,dx = (p+q)!/q!$, the normalisation factor can be derived as,

$$
N_{l,n} = \begin{cases}\sqrt{\frac{2n!}{\pi l! C(\sigma^{AB})^2}}, & l=n\\ \sqrt{\frac{n!}{\pi l! C^{l-n+1}(\sigma^{AB})^2}}, & \text{otherwise,}\end{cases} \quad (41)
$$

where the factor of 2 difference occurs for the case $l=n$, because the integral for the angular component is over $\cos\left(0\theta\right)$, a constant.

Separating the cos and sin terms in Eq. (39) since they are independent components, and letting

$$\alpha = \frac{(\sigma^{BC})^2}{2(\sigma^{AB})^2 + (\sigma^{BC})^2} \tag{42}$$

the eigenfunctions must satisfy

$$\begin{aligned} r^{l-n}\exp\left(-\frac{r^2}{2C}\right)\mathrm{L}_n^{l-n}\left(\frac{r^2}{C}\right)\cos\left((l-n)\theta\right) \\ \stackrel{?}{=} 2\pi A(\sigma^{AB})^2\exp\left(-\frac{r^2}{2\alpha}\right)\int_0^\infty d\tilde{r}\tilde{r}\exp\left(-\frac{\tilde{r}^2}{2\alpha}\right)\exp\left(\frac{-\tilde{r}^2}{2C}\right)\tilde{r}^{l-n}\mathrm{L}_n^{l-n}\left(\frac{\tilde{r}^2}{C}\right)I_{l-n}(r\tilde{r})\cos\left((l-n)\theta\right) \\ = 2\pi A(\sigma^{AB})^2\exp\left(-\frac{r^2}{2\alpha}\right)\int_0^\infty d\tilde{r}\tilde{r}\exp\left(-\frac{\tilde{r}^2}{2}\left(\frac{\alpha+C}{\alpha C}\right)\right)\tilde{r}^{l-n}I_{l-n}(r\tilde{r})\mathrm{L}_n^{l-n}\left(\frac{\tilde{r}^2}{C}\right)\cos\left((l-n)\theta\right). \end{aligned} \tag{43}$$

Note the following integral, evaluated using Wolfram Research Inc. (2018):

$$\begin{aligned} J_{n,l} &= \int_0^\infty d\tilde{r}\tilde{r}\exp\left(-\frac{\tilde{r}^2}{2B}\right)\tilde{r}^{l-n}I_{l-n}(r\tilde{r})\mathrm{L}_n^{l-n}\left(\frac{\tilde{r}^2}{C}\right) \\ &= B^{l+1}\left(\frac{C-2B}{BC}\right)^n\exp\left(\frac{Br^2}{2}\right)r^{l-n}\mathrm{L}_n^{l-n}\left(\frac{B^2r^2}{C-2B}\right). \end{aligned} \tag{44}$$

Applying this integral to Eq. (43) gives,

$$\begin{aligned} r^{l-n}\exp\left(-\frac{r^2}{2C}\right)\mathrm{L}_n^{l-n}\left(\frac{r^2}{C}\right)\cos\left((l-n)\theta\right) \\ \stackrel{?}{=} 2\pi A(\sigma^{AB})^2\exp\left(-\frac{r^2}{2\alpha}\right)\left(\frac{\alpha C}{\alpha+C}\right)^{l+1}\left(\frac{C-2\left(\frac{\alpha+C}{\alpha C}\right)}{\left(\frac{\alpha+C}{\alpha C}\right)C}\right)^n r^{l-n}\exp\left(\left(\frac{\alpha C}{\alpha+C}\right)\frac{r^2}{2}\right)\mathrm{L}_n^{l-n}\left(\frac{\left(\frac{\alpha+C}{\alpha C}\right)^2r^2}{C-2\left(\frac{\alpha+C}{\alpha C}\right)}\right)\cos\left((l-n)\theta\right) \\ = 2\pi A(\sigma^{AB})^2\left(\frac{C\alpha}{\alpha+C}\right)^{l+1}\left(\frac{C-\alpha}{C+\alpha}\right)^n r^{l-n}\exp\left(-\left(\frac{\alpha+C-2\alpha^2C}{\alpha(\alpha+C)}\right)\frac{r^2}{2}\right)\mathrm{L}_n^{l-n}\left(\frac{C\alpha^2r^2}{C^2-\alpha^2}\right)\cos\left((l-n)\theta\right). \end{aligned} \tag{45}$$

For this equivalence to be true, it is necessary to equate terms. After some simplification, it can be seen that equating both the exponential and Laguerre terms requires that,

$$\begin{aligned} \frac{1}{C} &= \frac{C\alpha^2}{C^2-\alpha^2} \\ \rightarrow 0 &= C(\alpha^2-1)+\alpha^2. \end{aligned} \tag{46}$$

Solving this quadratic in C requires,

$$C = \frac{\alpha}{\sqrt{1-\alpha^2}}, \tag{47}$$

from which condition we finally get,

$$\begin{aligned} r^{l-n}\exp\left(-\frac{r^2}{2C}\right)\mathrm{L}_n^{l-n}\left(\frac{r^2}{C}\right)\cos\left((l-n)\theta\right) \\ = 2\pi A(\sigma^{AB})^2\left(\frac{C\alpha}{\alpha+C}\right)^{l+n+1} r^{l-n}\exp\left(-\frac{r^2}{2C}\right)\mathrm{L}_n^{l-n}\left(\frac{r^2}{C}\right)\cos\left((l-n)\theta\right). \end{aligned} \tag{48}$$

Substituting the radial connection parameters back in using Eq. (42) finally gives,

$$C = \frac{(\sigma^{BC})^2}{2\sigma^{AB}\sqrt{(\sigma^{AB})^2+(\sigma^{BC})^2}}. \tag{49}$$

Consequently, the eigenfunctions and eigenvalues for the learning equation can be expressed in polar coordinates as,

$$\lambda_{l,n} = 2\pi A \left(\frac{C(\sigma^{BC})^2}{C((\sigma^{AB})^2 + (\sigma^{BC})^2) + (\sigma^{BC})^2} \right)^{l+n+1} \tag{50a}$$

$$\mathbf{v}_{l,n}(r,\theta) = N_{l,n} r^{l-n} \exp\left(-\frac{r^2}{2C}\right) \mathrm{L}_n^{l-n}\left(\frac{r^2}{C}\right) \exp\left(i(l-n)\theta\right). \tag{50b}$$

C.2 Full learning equation

If the simplified learning equation in Eq. (11) is denoted by H^0, from the eigenfunctions derived for the simplified learning equation, we know that,

$$\lambda^0_{l,n} \mathbf{v}^0_{l,n}(r,\theta) = \mathrm{H}^0 \mathbf{v}^0_{l,n}(r,\theta). \tag{51}$$

If we perturb the simplified learning equation by adding a small k_2^{BC}, denote the perturbed system by H, and the eigenfunctions of the perturbed system by $\mathbf{v}_{l,n}(r,\theta)$, where l and n determine the order of the eigenfunction, so that

$$\mathrm{H} = A \int_0^\infty d\tilde{r}\, \tilde{r} \int_0^{2\pi} d\tilde{\theta} \left(\exp\left(-\frac{r^2 + \tilde{r}^2 - 2r\tilde{r}\cos\left(\theta - \tilde{\theta}\right)}{2(\sigma^{AB})^2}\right) + k_2^{BC} \right) \exp\left(-\frac{\tilde{r}^2 + r^2}{(\sigma^{BC})^2}\right) w\left(\tilde{r},\tilde{\theta}\right), \tag{52}$$

and the eigenvectors for the full system, including the perturbation, satisfy

$$\lambda_{l,n} \mathbf{v}_{l,n}(r,\theta) = \mathrm{H} \mathbf{v}_{l,n}(r,\theta). \tag{53}$$

The perturbation on the integral operator, denoted H^1, is then,

$$\mathrm{H}^1 = A \int_0^\infty d\tilde{r}\, \tilde{r} \int_0^{2\pi} d\tilde{\theta}\, k_2^{BC} \exp\left(-\frac{\tilde{r}^2 + r^2}{(\sigma^{BC})^2}\right) \mathbf{v}^0_{l,n}(r,\theta), \tag{54}$$

and we require the new eigenfunctions to be similar to the eigenfunctions of the simplified learning equation, plus a small perturbation, so that the first order corrections to the eigenfunctions and eigenvalues can be defined as,

$$\mathbf{v}_{l,n}(r,\theta) = \mathbf{v}^0_{l,n}(r,\theta) + \mathbf{v}^1_{l,n}(r,\theta), \qquad \lambda_{l,n} = \lambda^0_{l,n} + \lambda^1_{l,n}. \tag{55}$$

If $W^{m,p}_{l,n} = \int_{-\infty}^{\infty} dr\, r \int_0^{2\pi} d\theta \left(\mathbf{v}^0_{l,n}(r,\theta)\right)^* \mathrm{H}^1\, \mathbf{v}^0_{m,p}(r,\theta)$, for non-degenerate eigenfunctions, , i.e. $l+n \neq m+p$ the first order corrections can be determined by (Kato, 1995)

$$\mathbf{v}^1_{l,n}(r,\theta) = \sum_{l+n \neq m+p} \frac{W^{m,p}_{l,n}}{\lambda^0_{l,n} - \lambda^0_{m,p}}, \mathbf{v}^0_{l,n}(r,\theta) \quad \text{and} \quad \lambda^1_{l,n} = W^{l,n}_{l,n}, \tag{56}$$

for non-degenerate eigenfunctions, i.e. $l+n \neq m+p$. For degenerate eigenfunctions, where the denominator of Eq. (56) is equal to zero, the first order correction of degenerate eigenfunctions of order $l+n$, can be found as a weighted sum of the degenerate eigenfunctions, where the weights are determined by the eigenvectors of the $(l+n) \times (l+n)$ matrix of $W^{m,p}_{l,n}$ coefficients. Given that the set of degenerate eigenfunctions of order $l+n$ have angular terms with different frequencies the off-diagonal terms of this matrix are 0. Hence, the eigenfunctions of this matrix are simply the terms, $W^{l,n}_{l,n}$. Additionally, where $l \neq n$, the diagonal terms will be zero because the perturbation integrates to 0 for each radii, which can be seen from the equal number of light and dark regions as you traverse from 0 to 2π at a given radius in Fig. 2. Consequently, the only non-zero perturbation term in a set of degenerate eigenfunctions are those for which $l = n$, which happens once, and only where $l+n$ is even.

For a pair of non-degenerate eigenfunctions where they have different angular frequencies the perturbation will also evaluate to zero. However, note that where the values $l-n$ equals $m-p$, so that the angular terms have the same frequency, some non-zero terms can appear. However, these terms are very small so can be ignored for the purposes of the perturbation

approximation. It is therefore only necessary to evaluate the diagonal terms, which are denoted by the single index pair, $W_{l,n}$, and can be evaluated as,

$$\begin{aligned} W_{l,n} &= 2\pi A N_{l,n}^2 \int_0^\infty d\tilde{r}\, \tilde{r}\, \tilde{r}^{2(l-n)} \exp\left(-\frac{r^2}{C}\right) \exp\left(-\frac{\tilde{r}^2+r^2}{(\sigma^{BC})^2}\right) \left[\mathrm{L}_n^{l-n}\left(\frac{r^2}{C}\right)\right]^2 \\ &= \pi A N_{l,n}^2 C^{l+n+1} \exp\left(-\frac{r^2}{(\sigma^{BC})^2}\right) \int_0^\infty dz\, z^{l-n} \exp(-\alpha z) \left[\mathrm{L}_n^{l-n}(z)\right]^2, \end{aligned} \tag{57}$$

where $\alpha = \frac{(\sigma^{BC})^2+C}{(\sigma^{BC})^2}$. This integral on the right hand side can be evaluated as (Gradshteyn and Ryzhik, 2007),

$$\int_0^\infty e^{-bx} x^a \mathrm{L}_n^a(x) \mathrm{L}_m^a(x)\, dx = \frac{\Gamma(m+n+a+1)(b-1)^{n+m}}{m!n!b^{m+n+a+1}} {}_2F_1\left(-m, -n; -m-n-a; \frac{b(b-2)}{(b-1)^2}\right), \tag{58}$$

where ${}_2F_1()$ is the hypergeometric function, and hence,

$$W_{l,n} = \pi C^{l-n+1} k_2^{BC} N_{l,n}^2 \frac{\Gamma(l+n+1)(\alpha-1)^{2n}}{n!^2 \alpha^{l+n+1}} {}_2F_1\left(-n, n; l-n; \frac{\alpha(\alpha-2)}{(\alpha-1)^2}\right). \tag{59}$$

Since only the diagonal terms are non-zero, the shape of the perturbed eigenfunctions remains the same as those for the simplified learning equation, given in Eq. (40), but the eigenvalues change according to

$$\lambda_{l,n} = \lambda_{l,n}^0 + W_{l,n}. \tag{60}$$

D Derivation of Cartesian eigenfunctions

To characterise learning in terms of the eigenfunctions it is useful to approximate the system in Eq. (5) by its continuous limit, and initially simplify the system by assuming that $k_2 = 0$. In this case we need to solve the eigenvalue problem that integrates the expected covariance over the layer, weighted by the probability of connection to the postsynaptic neuron, i, in layer C. That is, we need to solve the following eigenfunction equation,

$$\begin{aligned} \eta\lambda w(\mathbf{x}) =& A \int_{-\infty}^{\infty}\int_{-\infty}^{\infty} Q\left(f_j^B, f_i^B\right) \exp\left(-\frac{|\mathbf{x}'|^2}{(\sigma^{BC})^2}\right) \exp\left(-\frac{|\mathbf{x}|^2}{(\sigma^{BC})^2}\right) w\left(\mathbf{x}'\right) d^2\mathbf{x}' \\ =& A \int_{-\infty}^{\infty}\int_{-\infty}^{\infty} \exp\left(-\frac{|\mathbf{x}-\mathbf{x}'|^2}{2(\sigma^{AB})^2}\right) \exp\left(-\frac{|\mathbf{x}'|^2}{(\sigma^{BC})^2}\right) \exp\left(-\frac{|\mathbf{x}|^2}{(\sigma^{BC})^2}\right) w\left(\mathbf{x}'\right) d^2\mathbf{x}' \\ =& A \exp\left(-\left(x^2+y^2\right) \frac{2(\sigma^{AB})^2+(\sigma^{BC})^2}{2(\sigma^{AB})^2(\sigma^{BC})^2}\right) \\ & \int_{-\infty}^{\infty}\int_{-\infty}^{\infty} \exp\left(-\frac{(2(\sigma^{AB})^2+(\sigma^{BC})^2)\tilde{x}^2 + 2(\sigma^{BC})^2 x\tilde{x}}{2(\sigma^{AB})^2(\sigma^{BC})^2}\right) \exp\left(-\frac{(2(\sigma^{AB})^2+(\sigma^{BC})^2)\tilde{y}^2 + 2(\sigma^{BC})^2 y\tilde{y}}{2(\sigma^{AB})^2(\sigma^{BC})^2}\right) w\left(\mathbf{x}'\right) d\tilde{x} d\tilde{y}, \end{aligned} \tag{61}$$

where $w()$ is the continuous time approximation to w, and neuron m in layer B is denoted by its position vector $\mathbf{x} = (x, y)$, $\mathbf{x}' = (\tilde{x}, \tilde{y})$, and subscripts have been omitted for readability. The coefficient, A, contains the constant terms from the synaptic connection probability (Eq. (1)), such that

$$A = \left(\frac{1}{\pi(\sigma^{AB})^2}\right)^2. \tag{62}$$

Given the separability of the x and y dimensions, in conjunction with the exponential weight function, consider the Hermite polynomial as the form of the eigenfunction, such that the eigenfunctions are given by

$$\mathbf{v}_{u,v}\left(\frac{x}{\sqrt{C}}, \frac{y}{\sqrt{C}}\right) = N_{u,v} \mathrm{H}_u\left(\frac{x}{\sqrt{C}}\right) \mathrm{H}_v\left(\frac{y}{\sqrt{C}}\right) \exp\left(-\frac{x^2+y^2}{2C}\right), \tag{63}$$

where u and v denote the order of the polynomial for each dimension, giving a two-dimensional eigenfunction of order $u+v$, and $N_{u,v} = \frac{1}{\sqrt{2^u u!}}\frac{1}{\sqrt{2^v v!}}$ is a normalization constant (Roman, 1984). C is a parameter that must be determined. Consequently, when this expression is input to the eigenfunction equation from Eq. (61) the learning equation becomes,

$$\eta\lambda_{u,v}\mathbf{v}_{u,v}\left(\frac{x}{\sqrt{C}},\frac{y}{\sqrt{C}}\right) = A\exp\left(-\left(x^2+y^2\right)\frac{2(\sigma^{AB})^2+(\sigma^{BC})^2}{2(\sigma^{AB})^2(\sigma^{BC})^2}\right)\int\limits_{-\infty}^{\infty}\int\limits_{-\infty}^{\infty}\exp\left(-\frac{(2(\sigma^{AB})^2+(\sigma^{BC})^2)\tilde{x}^2+2(\sigma^{BC})^2x\tilde{x}}{2(\sigma^{AB})^2(\sigma^{BC})^2}\right)$$
$$\exp\left(-\frac{(2(\sigma^{AB})^2+(\sigma^{BC})^2)\tilde{y}^2+2(\sigma^{BC})^2y\tilde{y}}{2(\sigma^{AB})^2(\sigma^{BC})^2}\right)\mathbf{v}_{u,v}\left(\frac{\tilde{x}}{\sqrt{C}},\frac{\tilde{y}}{\sqrt{C}}\right)d\tilde{x}d\tilde{y}, \tag{64}$$

which holds true only when

$$C = \frac{(\sigma^{BC})^2}{2\sqrt{1+\frac{(\sigma^{BC})^2}{(\sigma^{AB})^2}}} = \frac{\sigma^{AB}(\sigma^{BC})^2}{2\sqrt{(\sigma^{AB})^2+(\sigma^{BC})^2}}, \tag{65}$$

which has units of distance, or $[C]=m^2$, that determines the spatial extent of synaptic projections in each dimension by scaling the inputs, x and y in the eigenfunctions. Note that this result agrees with the result derived for the radial eigenfunctions, Eq. (13), which gives a unitless expression for C, owing to scaling of r by $1/\sigma^{AB}$. If the input radius, r, was reverted back to distance via scaling by σ^{AB}, the expression for C in Eq. (13) is multiplied by $(\sigma^{AB})^2$, so that the expressions are be identical.

Due to the separability of the dimensions, the eigenvalues for x and y can be derived independently. Therefore initially consider the problem in just one dimension. In deriving the eigenvalues for the complete orthogonal set of Hermite polynomials, we follow the procedure used in (Wimbauer et al., 1998), and make the Ansatz that,

$$\lambda_u = \Lambda q^u. \tag{66}$$

Using the generating function for one dimensional Hermite polynomials,

$$\exp\left(-t^2+2t\frac{x}{\sqrt{C}}\right) = \sum_{l=0}^{\infty}\frac{t^l}{l!}\mathrm{H}_l\left(\frac{x}{\sqrt{C}}\right), \tag{67}$$

and the orthogonality of Hermite polynomials with respect to a Gaussian weight function, we know that,

$$\exp\left(-t^2+2t\frac{x}{\sqrt{C}}\right)\exp\left(-\frac{x^2}{2C}\right) = \sum_{l=0}^{\infty}\frac{t^l}{l!}\mathrm{H}_l\left(\frac{x}{\sqrt{C}}\right)\exp\left(-\frac{x^2}{2C}\right). \tag{68}$$

Combining this with Eq. (64), considering a single dimension only, gives,

$$\sum_{l=0}^{\infty}\lambda_l\frac{t^l}{l!}\mathrm{H}_l\left(\frac{x}{\sqrt{C}}\right)\exp\left(-\frac{x^2}{2C}\right) = \sqrt{A}\exp\left(-x^2\left(\frac{2(\sigma^{AB})^2+(\sigma^{BC})^2}{2(\sigma^{AB})^2(\sigma^{BC})^2}\right)\right)$$
$$\int_{-\infty}^{\infty}\exp\left(-\frac{\left((2(\sigma^{AB})^2+(\sigma^{BC})^2\right)\tilde{x}^2+2(\sigma^{BC})^2\tilde{x}x)}{2(\sigma^{AB})^2(\sigma^{BC})^2}\right)\sum_{l=0}^{\infty}\frac{t^l}{l!}\mathrm{H}_l\left(\frac{\tilde{x}}{\sqrt{C}}\right)\exp\left(-\frac{x^2}{2C}\right)d\tilde{x}$$
$$= \sqrt{A}\exp\left(-x^2\left(\frac{2(\sigma^{AB})^2+(\sigma^{BC})^2}{2(\sigma^{AB})^2(\sigma^{BC})^2}\right)\right)$$
$$\int_{-\infty}^{\infty}\exp\left(-\frac{\left((2(\sigma^{AB})^2+(\sigma^{BC})^2\right)\tilde{x}^2+2(\sigma^{BC})^2\tilde{x}x)}{2(\sigma^{AB})^2(\sigma^{BC})^2}\right)\exp\left(-t^2+2t\frac{\tilde{x}}{\sqrt{C}}\right)\exp\left(-\frac{x^2}{2C}\right)d\tilde{x} \tag{69}$$

Evaluating the right hand side,

$$
\begin{aligned}
\text{RHS} &= \sqrt{A}\exp\left(-x^2\left(\frac{2(\sigma^{AB})^2+(\sigma^{BC})^2}{2(\sigma^{AB})^2(\sigma^{BC})^2}\right)\right) \\
&\quad \int_{-\infty}^{\infty}\exp\left(-\frac{\left(\left(2C(\sigma^{AB})^2+C(\sigma^{BC})^2+(\sigma^{AB})^2(\sigma^{BC})^2\right)\tilde{x}^2+2C(\sigma^{BC})^2\tilde{x}x\right)}{2C(\sigma^{AB})^2(\sigma^{BC})^2}\right)\exp\left(-t^2+2t\frac{\tilde{x}}{\sqrt{C}}\right)d\tilde{x} \\
&= \sqrt{A}\exp\left(-t^2\right)\exp\left(-x^2\left(\frac{2(\sigma^{AB})^2+(\sigma^{BC})^2}{2(\sigma^{AB})^2(\sigma^{BC})^2}\right)\right) \\
&\quad \int_{-\infty}^{\infty}\exp\left(-\frac{\left(2C(\sigma^{AB})^2+C(\sigma^{BC})^2+(\sigma^{AB})^2(\sigma^{BC})^2\right)\tilde{x}^2+\left(2C(\sigma^{BC})^2x+4t\sqrt{C}(\sigma^{AB})^2(\sigma^{BC})^2\right)\tilde{x}}{2C(\sigma^{AB})^2(\sigma^{BC})^2}\right)d\tilde{x} \\
&= \sqrt{A}\exp\left(-t^2\right)\exp\left(-x^2\left(\frac{2(\sigma^{AB})^2+(\sigma^{BC})^2}{2(\sigma^{AB})^2(\sigma^{BC})^2}\right)\right)\exp\left(\frac{\left(C(\sigma^{BC})^2x+2t\sqrt{C}(\sigma^{AB})^2(\sigma^{BC})^2\right)^2}{\left(2C(\sigma^{AB})^2+C(\sigma^{BC})^2+(\sigma^{AB})^2(\sigma^{BC})^2\right)2C(\sigma^{AB})^2(\sigma^{BC})^2}\right) \\
&\quad \int_{-\infty}^{\infty}\exp\left(-\frac{1}{2C(\sigma^{AB})^2(\sigma^{BC})^2}\left(\left(2C(\sigma^{AB})^2+C(\sigma^{BC})^2+(\sigma^{AB})^2(\sigma^{BC})^2\right)^{\frac{1}{2}}\tilde{x}-\frac{C(\sigma^{BC})^2x+2t\sqrt{C}(\sigma^{AB})^2(\sigma^{BC})^2}{(2C(\sigma^{AB})^2+C(\sigma^{BC})^2+(\sigma^{AB})^2(\sigma^{BC})^2)^{\frac{1}{2}})}\right)^2\right)d\tilde{x} \\
&= \sqrt{A}\exp\left(-t^2\right)\left(\frac{2\pi C(\sigma^{AB})^2(\sigma^{BC})^2}{2C(\sigma^{AB})^2+C(\sigma^{BC})^2+(\sigma^{AB})^2(\sigma^{BC})^2}\right) \\
&\quad \exp\left(-x^2\left(\frac{2(\sigma^{AB})^2+(\sigma^{BC})^2}{2(\sigma^{AB})^2(\sigma^{BC})^2}\right)\right)\exp\left(\frac{\left(C(\sigma^{BC})^2x+2t\sqrt{C}(\sigma^{AB})^2(\sigma^{BC})^2\right)^2}{\left(2C(\sigma^{AB})^2+C(\sigma^{BC})^2+(\sigma^{AB})^2(\sigma^{BC})^2\right)2C(\sigma^{AB})^2(\sigma^{BC})^2}\right). \qquad (70)
\end{aligned}
$$

Evaluating the left hand side of Eq. (69) gives

$$
\begin{aligned}
\text{LHS} &= \sum_{l=0}^{\infty}\Lambda_0\frac{(qt)^l}{l!}\text{H}_l\left(\frac{x}{\sqrt{C}}\right)\exp\left(-\frac{x^2}{2C}\right) \\
&= \Lambda_0\exp\left(-(qt)^2+\frac{2q}{\sqrt{C}}xt\right)\exp\left(-\frac{x^2}{2C}\right). \qquad (71)
\end{aligned}
$$

Comparing RHS and LHS requires that

$$
q = \frac{C(\sigma^{BC})^2}{2(\sigma^{AB})^2C+(\sigma^{BC})^2C+(\sigma^{AB})^2(\sigma^{BC})^2}, \qquad (72)
$$

and

$$
\begin{aligned}
\Lambda_0 &= \sqrt{\frac{2\pi C(\sigma^{AB})^2(\sigma^{BC})^2}{2C(\sigma^{AB})^2+2C(\sigma^{BC})^2+(\sigma^{AB})^2(\sigma^{BC})^2}} \\
&= \sqrt{2\pi(\sigma^{AB})^2q} \qquad (73)
\end{aligned}
$$

so that

$$
\begin{aligned}
\lambda_l &= \Lambda_0 q^l \\
&= \sqrt{2\pi(\sigma^{AB})^2}q^{l+\frac{1}{2}}. \qquad (74)
\end{aligned}
$$

For two dimensions, the final eigenfunctions and eigenvalue pairs, for order u and v, for the x and y dimensions respectively, are given by,

$$
\lambda_{u,v} = 2\pi(\sigma^{AB})^2q^{u+v+1} \qquad (75a)
$$

$$
\mathbf{v}_{u,v}\left(\frac{x}{\sqrt{C}},\frac{y}{\sqrt{C}}\right) = \frac{1}{\sqrt{2^u u!}}\frac{1}{\sqrt{2^v v!}}\text{H}_u\left(\frac{x}{\sqrt{C}}\right)\text{H}_v\left(\frac{y}{\sqrt{C}}\right)\exp\left(-\frac{x^2+y^2}{2C}\right). \qquad (75b)
$$

E Expected number of shared inputs with radially dependent cell density

We consider the expected number of shared inputs between two cells in layer B when there is radially dependent cell density, such that cell density is highest in the centre of the layer, and decreases linearly with distance from the centre. In this case, an assumed consequence is a reduction in the radius of the synaptic connectivity distribution in the layer centre, with a corresponding linear increase with radial distance traversed from the layer centre.

The probability of presynaptic neuron, m, in layer A, generating a synaptic connection to postsynaptic neuron, i, in layer B, is given in Eq. (1).

If neuron j is d_{jc}^{B} far from the layer centre, with a cell connection density of σ^{i}, then the expression for the expected number of neurons in layer A connecting to both neuron i and neuron j, in the continuous limit, is given by

$$\begin{aligned}
\mathrm{E}\left[N^{AB};[x_i,y_j],[x_i,y_j]\right] &= \frac{(N^{AB})^2}{\pi^2\,(\sigma^{AB})^4\,d_i^2 d_j^2}\iint\limits_{xy}\exp\left(-\frac{(x-x_i)^2+(y-y_i)^2}{d_i^2(\sigma^{AB})^2}\right)\exp\left(-\frac{(x-x_j)^2+(y-y_j)^2}{d_j^2(\sigma^{AB})^2}\right)dx\,dy,\\
&= \frac{(N^{AB})^2}{\pi^2\,(\sigma^{AB})^4\,d_i^2 d_j^2}\int\limits_{x}\exp\left(-\frac{d_j^2\,(x-x_i)^2+d_i^2\,(x-x_j)^2}{d_j^2 d_i^2(\sigma^{AB})^2}\right)dx\int\limits_{y}\exp\left(-\frac{d_j^2\,(y-y_i)^2+d_i^2\,(y-y_j)^2}{d_i^2 d_j^2(\sigma^{AB})^2}\right)dy,
\end{aligned} \tag{76}$$

where we drop the centre subscript, c, and layer superscript, ^{BB}, for readability. The exponent in the left sum can be simplified as

$$\begin{aligned}
&\frac{d_j^2\,(x-x_i)^2+d_i^2\,(x-x_j)^2}{d_j^2 d_i^2(\sigma^{AB})^2}\\
&\qquad= \frac{d_i^2+d_j^2}{d_i^2 d_j^2(\sigma^{AB})^2}\left[x^2-\frac{2\left(d_j^2 x_i+d_i^2 x_j\right)}{d_i^2+d_j^2}x+\frac{d_j^2 x_i^2+d_i^2 x_j^2}{d_i^2+d_j^2}\right]\\
&\qquad= \frac{d_i^2+d_j^2}{d_i^2 d_j^2(\sigma^{AB})^2}\left[\left(x-\frac{d_j^2 x_i+d_i^2 x_j}{d_i^2+d_j^2}\right)^2-\left(\frac{d_j^2 x_i+d_i^2 x_j}{d_i^2+d_j^2}\right)^2+\frac{d_j^2 x_i^2+d_i^2 x_j^2}{d_i^2+d_j^2}\right]\\
&\qquad= \frac{d_i^2+d_j^2}{d_i^2 d_j^2(\sigma^{AB})^2}\left[\left(x-\frac{d_j^2 x_i+d_i^2 x_j}{d_i^2+d_j^2}\right)^2-\frac{d_j^4 x_i^2+d_i^4 x_j^2+2d_i^2 d_j^2 x_i x_j}{\left(d_i^2+d_j^2\right)^2}+\frac{d_j^2 x_i^2+d_i^2 x_j^2}{d_i^2+d_j^2}\right]\\
&\qquad= \frac{d_i^2+d_j^2}{d_i^2 d_j^2(\sigma^{AB})^2}\left[\left(x-\frac{d_j^2 x_i+d_i^2 x_j}{d_i^2+d_j^2}\right)^2-\frac{\left(d_j^2+d_j^2\right)^2\left(d_j^2 x_i^2+d_i^2 x_j^2\right)+2d_i^2 d_j^2 x_i x_j-d_i^2 d_j^2 x_i-d_i^2 d_j^2 x_j}{\left(d_i^2+d_j^2\right)^2}+\frac{d_j^2 x_i^2+d_i^2 x_j^2}{d_i^2+d_j^2}\right]\\
&\qquad= \frac{d_i^2+d_j^2}{d_i^2 d_j^2(\sigma^{AB})^2}\left[\left(x-\frac{d_j^2 x_i+d_i^2 x_j}{d_i^2+d_j^2}\right)^2-\frac{d_j^2 x_i^2+d_i^2 x_j^2}{d_i^2+d_j^2}+\frac{d_j^2 x_i^2+d_i^2 x_j^2}{d_i^2+d_j^2}-\frac{d_i^2 d_j^2\left(x_i^2+x_j^2-2x_i x_j\right)}{\left(d_i^2+d_j^2\right)^2}\right]\\
&\qquad= \frac{d_i^2+d_j^2}{d_i^2 d_j^2(\sigma^{AB})^2}\left(x-\frac{d_j^2 x_i+d_i^2 x_j}{d_i^2+d_j^2}\right)^2-\left(\frac{d_i^2+d_j^2}{d_i^2 d_j^2(\sigma^{AB})^2}\right)\frac{d_i^2 d_j^2\left(x_i^2+x_j^2-2x_i x_j\right)}{\left(d_i^2+d_j^2\right)^2}\\
&\qquad= \frac{d_i^2+d_j^2}{d_i^2 d_j^2(\sigma^{AB})^2}\left(x-\frac{d_j^2 x_i+d_i^2 x_j}{d_i^2+d_j^2}\right)^2-\frac{(x_i-x_j)^2}{(\sigma^{AB})^2\left(d_i^2+d_j^2\right)}.
\end{aligned}$$

Applying this result to the y integrand in Eq. (76) also, we get

$$\begin{aligned}
\mathrm{E}\left[N^{AB};[x_i,y_i],[x_i,y_i]\right] =& \frac{(N^{AB})^2}{\pi^2(\sigma^{AB})^4 d_i^2 d_j^2}\int_x \exp\left(-\frac{d_i^2+d_j^2}{d_i^2 d_j^2(\sigma^{AB})^2}\left(x-\frac{d_j^2 x_i+d_i^2 x_j}{d_i^2+d_j^2}\right)^2-\frac{(x_i-x_j)^2}{(\sigma^{AB})^2\left(d_i^2+d_j^2\right)}\right)dx \\
&\int_y \exp\left(-\frac{d_i^2+d_j^2}{d_i^2 d_j^2(\sigma^{AB})^2}\left(y-\frac{d_j^2 y_i+d_i^2 y_j}{d_i^2+d_j^2}\right)^2-\frac{(y_i-y_j)^2}{(\sigma^{AB})^2\left(d_i^2+d_j^2\right)}\right)dy \\
=& \frac{(N^{AB})^2}{\pi(\sigma^{AB})^4 d_i^2 d_j^2}\exp\left(-\frac{(x_i-x_j)^2+(y_i-y_j)^2}{(\sigma^{AB})^2\left(d_i^2+d_j^2\right)}\right)\left(\frac{\pi(\sigma^{AB})^2 d_i^2 d_j^2}{d_i^2+d_j^2}\right)^{1/2}\left(\frac{\pi(\sigma^{AB})^2 d_i^2 d_j^2}{d_i^2+d_j^2}\right)^{1/2} \\
=& \frac{(N^{AB})^2}{\pi(\sigma^{AB})^2\left(d_i^2+d_j^2\right)}\exp\left(-\frac{(x_i-x_j)^2+(y_i-y_j)^2}{(\sigma^{AB})^2\left(d_i^2+d_j^2\right)}\right) \\
=& \frac{(N^{AB})^2}{\pi(\sigma^{AB})^2\left(d_i^2+d_j^2\right)}\exp\left(-\frac{d_{ij}^2}{(\sigma^{AB})^2\left(d_i^2+d_j^2\right)}\right) \qquad (77)
\end{aligned}$$

References

P. Antinucci and R. Hindges. Orientation-selective retinal circuits in vertebrates. *Frontiers in neural circuits*, 12:11, 2018.

E. L. Bienenstock, L. N. Cooper, and P. W. Munro. Theory for the development of neuron selectivity: Orientation specificity and binocular interaction in visual cortex. *The Journal of Neuroscience*, 2(1):32–38, 1982.

C. E. Davey, D. B. Grayden, and A. N. Burkitt. Impact of axonal delay on structure development in a multi-layered network. *Neural Networks*, 144:737–754, 2021.

J. Freeman, G. J. Brouwer, D. J. Heeger, and E. P. Merriam. Orientation decoding depends on maps, not columns. *Journal of Neuroscience*, 31(3):4792–4804, 2011.

W Gerstner, R. Kempter, J. L. van Hemmen, and H. Wagner. A neuronal learning rule for sub-millisecond temporal coding. *Nature*, 383:76–78, 1996.

G. Goodhill. Contributions of theoretical modeling to the understanding of neural map development. *Neuron*, 56:301–311, 2007.

I. S. Gradshteyn and I. M Ryzhik. *Table of integrals, series and products*, chapter 7, page 810. Elsevier, 7th edition, 2007.

T. Kato. *Perturbation Theory for Linear Operators*, chapter 6, pages 1–643. Springer, second edition, 1995.

R. Kempter, W. Gerstner, and J. L. van Hemmen. Hebbian learning and spiking neurons. *Physical Review E*, 59(4):4498–4514, 1999a.

R. Kempter, W. Gerstner, and J. L. van Hemmen. Spike-based compared to rate-based Hebbian learning. *Advances in Neuron Information Processing Systems*, 11:128–131, 1999b.

R. Linsker. From basic network principles to neural architecture: Emergence of spatial-opponent cells. *Proceedings of the National Academy of Sciences, USA*, 83:7508–7512, 1986a.

R. Linsker. From basic network principles to neural architecture: Emergence of orientation-selective cells. *Proceedings of the National Academy of Sciences, USA*, 83:8390–8394, 1986b.

D. J. MacKay and K. D. Miller. Analysis of Linsker's application of Hebbian rules to linear networks. *Network: Computation in Neural Systems*, 1:257–297, 1990.

I. Mareschal, S. C. Dakin, and P. J. Bex. Dynamic properties of orientation discrimination assessed by using classification images. In *Proceedings of the National Academy of Sciences*, volume 103(13, pages 5131–5136, 2006.

H. Markram, J. Lubke, M. Frotscher, and B. Sakmann. Regulation of synaptic efficacy by coincidence of postsynaptic aps and epsps. *Science*, 275:213–215, 1997.

K. D. Miller. Correlation-based models of neural development. In M. A. Gluck and D. E. Rumelhart, editors, *Neuroscience and Connectionist Theory*, chapter 7, pages 267–352. Hillsdale, 1990.

R. T. Philips and V. S. Chakravarthy. A global orientation map in the primary visual cortex (V1): Could a self organizing model reveal its hidden bias? *Frontiers in neural circuits*, 10:109, 2017.

E. I. Rodionova, A. V. Revishchin, and I. N. Pigarev. Distant cortical locations of the upper and lower quadrants of the visual field represented by neurons with elongated and radially oriented receptive fields. *Experimental Brain Research*, 158:373–377, 2004.

S. Roman, editor. *The Umbral Calculus. Pure and Applied Mathematics*. Academic Press, 1st edition, 1984.

Y. Sasaki, R. Rajimehr, B. W. Kim, L. B. Ekstrom, W. Vanduffel, and R. B. Tootell. The radial bias: a different slant on visual orientation sensitivity in human and nonhuman primates. *Neuron*, 51(5):661–670, 2006.

B. Scholl, A. Y. Tan, J. Corey, and N. J. Priebe. Emergence of orientation selectivity in the mammalian visual pathway. *Journal of Neuroscience*, 33(26):10616–10624, 2013.

W. Senn and N. J. Buchs. Spike-based synaptic plasticity and the emergence of direction selective simple cells: Mathematical analysis. *Journal of Computational Neuroscience*, 14:119–138, 2003.

J. Sjöstrand, V. Olsson, Z. Popovic, and N. Conradi. Quantitative estimations of foveal and extra-foveal circuitry in humans. *Vision Research*, 39(18):2987–2998, 1999.

A. T. Smith, K. D. Singh, A. L. Williams, and M. W. Greenlee. Estimating receptive field size from fMRI data in human striate and extrastriate visual cortex. *Cerebral Cortex*, 11:1182–1190, 2001.

D. S. Tang. *Self-organization, emerging properties, and learning*, chapter Information theory and early Visual information processing, pages 113–125. Plenum Press, New York, 1st edition, 1990.

L. Walton and D. L. Bissest. Parameterising feature sensitive cell formation in Linsker's networks in the auditory system. In *Neural Information Processing Systems*, pages 1007–1013, 1992.

A. B. Watson. A formula for human retinal ganglion cell receptive field density as a function of visual field location. *Journal of Vision*, 14(7):15, 2014.

S. Wimbauer, O. G. Wenisch, K. D. Miller, and J. L. van Hemmen. Development of spatiotemporal receptive fields of simple cells: I. Model formulation. *Biological Cybernetics*, 77:453–461, 1997a.

S. Wimbauer, O. G. Wenisch, K. D. Miller, and J. L. van Hemmen. Development of spatiotemporal receptive fields of simple cells: II. Simulation and analysis. *Biological Cybernetics*, 77:463–477, 1997b.

S. Wimbauer, W. Gerstner, and J. L. van Hemmen. Analysis of a correlation-based model for the development of orientation-selective receptive fields in the visual cortex. *Network: Computation in Neural Systems*, 9:449–466, 1998.

Wolfram Research Inc. Mathematica, Version 11.3, 2018. Champaign, IL, 2018.

J. Wurbs, E. Mingolla, and A. Yazdanbakhsh. Modeling a space-variant cortical representation for apparent motion. *Journal of Vision*, 13(10):1–17, 2013.

T. Yamakazi. A mathematical analysis of the development of oriented receptive fields in Linsker's model. *Neural Networks*, 15:201–207, 2002.
